# Integrated-gut-liver-on-a-chip platform as an in vitro human model of non-alcoholic fatty liver disease

**DOI:** 10.1038/s42003-023-04710-8

**Published:** 2023-03-23

**Authors:** Jiandong Yang, Yoshikazu Hirai, Kei Iida, Shinji Ito, Marika Trumm, Shiho Terada, Risako Sakai, Toshiyuki Tsuchiya, Osamu Tabata, Ken-ichiro Kamei

**Affiliations:** 1 Department of Micro Engineering, Kyoto University, Kyotodaigaku-Katsura, Nishikyo-ku, Kyoto, 615-8540 Japan; 2 Department of Mechanical Engineering and Science, Kyoto University, Kyotodaigaku-Katsura, Nishikyo-ku, Kyoto, 615-8540 Japan; 3 Medical Research Support Center, Graduate School of Medicine, Kyoto University, Yoshida Konoe-cho, Kyoto, 606-8501 Japan; 4 Faculty of Science and Engineering, Kindai University, 3-4-1 Kowakae, Higashiosaka, Osaka, 577-8502 Japan; 5 Institute for Integrated Cell-Material Sciences, Kyoto University, Yoshida-Ushinomiya-cho, Sakyo-ku, Kyoto, 606-8501 Japan; 6 Institute for Pharmacy and Molecular Biotechnology, Heidelberg University, Heidelberg, 69120 Germany; 7 Faculty of Engineering/Graduate School of Engineering, Kyoto University of Advanced Science, Gotanda-cho, Yamanouchi, Ukyo-ku, Kyoto, 615-8577 Japan; 8 Wuya College of Innovation, Shenyang Pharmaceutical University, 110016 Liaoning, China; 9 Department of Pharmaceutics, Shenyang Pharmaceutical University, 110016 Liaoning, China; 10 Programs of Biology and Bioengineering, Divisions of Science and Engineering, New York University Abu Dhabi, Abu Dhabi, UAE

**Keywords:** Gastrointestinal models, Assay systems, Lab-on-a-chip, Cell culture

## Abstract

Non-alcoholic fatty liver disease (NAFLD) afflicts a significant percentage of the population; however, no effective treatments have yet been established because of the unsuitability of in vitro assays and animal experimental models. Here, we present an integrated-gut-liver-on-a-chip (iGLC) platform as an in vitro human model of the gut-liver axis (GLA) by co-culturing human gut and liver cell lines interconnected via microfluidics in a closed circulation loop, for the initiation and progression of NAFLD by treatment with free fatty acids (FFAs) for 1 and 7 days, respectively. Co-cultured Caco-2 gut-mimicking cells and HepG2 hepatocyte-like cells demonstrate the protective effects from apoptosis against FFAs treatment, whereas mono-cultured cells exhibit induced apoptosis. Phenotype and gene expression analyses reveal that the FFAs-treated gut and liver cells accumulated intracellular lipid droplets and show an increase in gene expression associated with a cellular response to copper ions and endoplasmic reticulum stress. As an in vitro human GLA model, the iGLC platform may serve as an alternative to animal experiments for investigating the mechanisms of NAFLD.

## Abstract (web summary)

A model system for studying non-alcoholic fatty liver disease is created by co-culturing human gut and liver cell lines in a closed circulation loop using microfluidics.

## Introduction

Non-alcoholic fatty liver disease (NAFLD) is a common, chronic liver disease that leads to hepatic steatosis, cirrhosis, cancer, and cardiovascular diseases^1–4^. NAFLD is expected to afflict 33.5% of the United States’ population over 15 years of age by 2030^5^. Currently, liver transplantation is the only method to cure patients with severe liver diseases, and finding donors that match patients is extremely difficult. There is an urgent need for intervention at different stages of fatty liver disease; however, the disease mechanism is largely unknown due to the complicated processes that take place at multiple layers, known as the multiple-hit theory. For example, fat accumulation, oxidative stress, endoplasmic reticulum (ER) stress, and genetic or epigenetic modifications can occur at the cellular level, while insulin resistance and inflammatory responses can occur in multiple organs depending on the individual and environment^6^. To identify new treatments for NAFLD, a deep understanding of each of these processes is needed, and this accumulated knowledge then needs to be combined.

In this study, we focused on the gut-liver axis (GLA), which is one of the most crucial components for the initiation and progression of NAFLD^7,8^. The gut is strongly influenced by gut microbiota and dietary carbohydrates, which may accelerate NAFLD^9–11^. Inflammatory products, nutrients, and substances absorbed from food and microbiota via the intestinal barrier are carried by venous blood to the liver. In addition, the products generated by the hepatocytes are transported to the small intestine. Thus, the gut and liver are intricately linked both physiologically and pathologically. GLA dysfunction, including intestinal dysbiosis, bacterial overgrowth, and alteration of mucosa permeability, caused by NAFLD, are potential therapeutic targets;^12,13^ but no treatments have been made commercially available to date. This is largely because conventional preclinical animal tests do not accurately represent the problems of the multiple-hit theory, lack accessibility to individual organs in living animals, and have species differences. Therefore, establishing a simplified and robust model to study GLA in NAFLD is crucial to obtaining deeper insights into the mechanisms underlying the discovery of new drugs, treatments, and diagnostic tools.

Organs-on-chips (OOCs), which are also known as microphysiological systems (MPSs), hold significant potential for in vitro preclinical tests^14–17^ and disease modeling^18^. Microfluidic technology is the foundation of OOCs because it enables precise control of liquid flow and the three-dimensional architecture of flow channels. These properties give OOCs the ability to control cellular microenvironments spatiotemporally and functionalize tissue cells. The circulation of the cell culture medium can further assist in modeling multi-organ interactions with paracrine and endocrine signaling. OCCs in combination with advanced cellular assays, such as high-content analyses and the omics approach, provide more in-depth insights into biology in a quantitative and multi-parametric manner than animal experiments^19^. OOCs have been used to recapitulate GLA in vitro and to demonstrate the role of crosstalk via the GLA in pathological situations, including fatty liver disease^20,21^ and inflammation^22^, for in vitro pharmacokinetic studies^23^. However, OOCs need to be further improved to mimic the GLA, and this requires four key features: a closed circulation loop, accessibility to individual chambers, dynamic flow control, and prevention of molecule absorption. The closed circulation loop is necessary for medium circulation to recapitulate inter-tissue interactions in the GLA. Individual accessibility is necessary to introduce tissue cells into the desired chamber and harvest them after treatment without cross-contamination from other cells. The closed circulation loop and individual accessibility may appear to be contradictory features, but both are necessary to investigate the crosstalk between the gut and liver. Dynamic flow control is crucial for obtaining functional tissue cells in vitro, particularly for the gut^24^. Some OOCs require the use of additional cell culture inserts (e.g., Transwell) to co-culture two or more types of cells separated by a porous membrane. However, due to the macroscale range of the medium, these inserts cannot be utilized for microfabrication and often lack the advantages of microfluidic technology, such as control over the flow dynamics in the cell culture chamber and the cellular microenvironment. These additional inserts often interfere with the microscopic observation of cells as they increase the working distance and light diffraction by the membrane pores. Polydimethylsiloxane (PDMS) is a widely used material for microfluidic cell culture systems due to its biocompatibility, transparency, and elasticity properties. However, preventing PDMS absorption is necessary, as PDMS causes the absorption of hydrophobic molecules, including metabolites, hormones, drug candidates, fatty acids, lipids, and fluorescent indicators, which may influence cellular phenotypes and assay results. Free fatty acids (FFAs) are a critical factor in NAFLD. Although PDMS-based microfluidic cell culture systems^25,26^, including our previously reported in vitro GLA model^27^, solve the aforementioned issues of OOCs for GLA, PDMS-based platforms without any treatments for preventing the absorption of hydrophobic molecules are not applicable for recapitulating NAFLD^28^.

Here, we present an integrated-gut-liver-on-a-chip (iGLC) platform as a simplified in vitro human model of GLA, which can help obtain deeper insights into the underlying mechanisms of NAFLD for the development of new drugs, treatments, and diagnostic tools. The iGLA platform^29^ has microvalves and a pump to achieve individual accessibility for each cell culture chamber and a closed medium circulation flow to interconnect the gut and liver cells. The integrated micropump controls the perfusion flow to activate cultured gut cells. The iGLC platform does not require additional cell culture inserts and therefore allows high-quality cell monitoring to achieve microscopic single-cell profiling. A simple surface coating with amphipathic molecules prevents the absorption of FFAs into PDMS. We co-cultured gut and liver cells with a closed circulation flow to demonstrate the viability of the iGLC platform as an in vitro human GLA model. We also induced an NAFLD-like cellular state by administering FFAs into the platform for two durations (1 and 7 days) to represent the initial and progressive NAFLD. Finally, we investigated the unique cellular phenotypic changes and associated gene networks for the GLA in the NAFLD-like cellular state by microscopic single-cell profiling in combination with mRNA sequencing (mRNA-seq).

## Results

### Fabrication of the iGLC platform

**Fig. 1:**
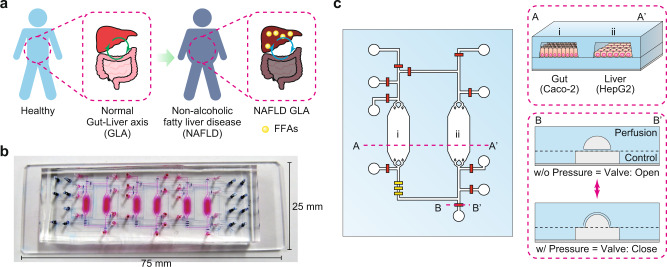
Design of the iGLC platform to recapitulate NAFLD. **a** Schematic for NAFLD progression by FFAs via the GLA. **b** Photograph of an iGLC platform. The perfusion and control layers are colored in pink and blue, respectively. **c** Illustration of the iGLC platform used for NAFLD. Two cell culture chambers are used for gut cells (Caco-2) and hepatocytes (HepG2) and are linked via a microfluidic channel with a micropump to achieve closed medium circulation of FFAs. Within the microvalves, each cell culture chamber is individually accessible without the risk of cross-contamination. Thus, this setup allows the inter-tissue interaction to be evaluated. A–A’ shows the cross-section of the cell culture chambers for the Caco-2 gut and HepG2 liver cells. B–B’ shows the cross-sectional view of the open and closed integrated microvalves. Because of the elastic PDMS membrane, the normally open valve is closed by applying higher pressure to the microfluidic channel in the control layer.

A conceptual illustration of the iGLC platform is shown in Fig. 1a. The design of the microfluidic platform is critical for recapitulating the GLA in vitro. Models based on animal experiments have difficulty investigating the mechanism of NAFLD progression because living organs cannot be connected and disconnected to make simplified multiple-hit-theory models. To elucidate inter-tissue interactions, a single platform is needed that can both mono- and co-culture two or more cell types within the same format. In parallel, cross-contamination needs to be considered to allow the analysis of each cell type and the influence from other cell types. We designed the iGLC platform to meet these requirements (Fig. 1b, c)^29^. The iGLC platform is made of PDMS with gas permeability, biocompatibility, and transparency and consists of perfusion and control layers. The perfusion layer had three sets of two cell culture chambers (2.1 mm width and 220 µm height) linked by microfluidic channels (200 µm width and 45 µm height). The control layer had a thin and flexible PDMS membrane (200 × 200 µm^2^ area and 20 µm thickness) to integrate the microvalves and pump, which allowed robust open/close control with our previously reported microfabrication technology (Supplementary Fig. S1)^29,30^. To investigate the interactions between two or more types of tissue cells, it is necessary to isolate and connect them as desired. Because most OOCs do not have integrated valves and pumps, they require assembling and disassembling tubes among multiple devices, which reduces the accuracy of the control over their interactions, but avoids undesired cross-contamination. The integrated micropump provides more accurate control over the perfused flow than an external pump within the microfluidic device. In addition, it reduces the sample loss caused by extra tubing. The PDMS membranes at the microvalves and pump were actuated by a positive hydraulic pressure (150 kPa) applied by the computer-controlled solenoid valves. The microvalves allow individual cell culture chambers to be accessed without cross-contamination and precise control over sample or reagent introduction. The integrated micropump provides closed-loop medium circulation to recapitulate inter-tissue interactions, such as the GLA, and to regulate the flow rate within 0–20 nL min^−1^ by tuning its actuating cycle (Supplementary Fig. S2).

### Prevention of absorption of hydrophobic molecules in PDMS

Prior to the cell culture and FFAs treatments, we coated the PDMS surface of the cell culture chambers with *n*-dodecyl β-D-maltoside (DDM)^31,32^ and then Matrigel to reduce the absorption of hydrophobic molecules (e.g., FFAs and AdipoRed fluorescent lipid marker)^33,34^ and to increase cell adhesion and growth (Supplementary Fig. S3). This is a critical problem when using PDMS-based OOCs for drug discovery and in vitro disease modeling because small-molecule drug candidates and lipids are absorbed by the PDMS before reaching the target cells, and the concentrations differ from those in a microtiter plate. Under these conditions, the results obtained would be unreliable. DDM is a suitable amphipathic molecule that prevents PDMS absorption because its hydrophobic part binds to the PDMS surface and the hydrophilic part prevents the absorption of hydrophobic molecules. However, DDM is not capable of promoting cell adhesion and growth on the PDMS surface. Therefore, we evaluated combining DDM with a subsequent layer of Matrigel. The DDM/Matrigel coating partially prevented the absorption of the AdipoRed fluorescent lipid marker into the PDMS (Supplementary Fig. S4). We also measured the remaining FFAs [a mixture of palmitic acid (PA) and oleic acid (OA) with a molar ratio of 1:2; see Methods] in the cell culture medium after incubation with the coated PDMS (Supplementary Fig. S5). PA was not absorbed by either the non-coated or DDM/Matrigel-coated PDMS after 6 h of incubation at 37 °C, whereas it showed absorptions of 53.7% and 40.9%, respectively, after 24 h of incubation. OA showed absorptions of 22.0% and 17.4%, respectively, after 6 h of incubation, and absorptions of 67.4% and 56.7%, respectively, after 24 h of absorption. These results suggest that although the DDM/Matrigel coating slightly mitigated the absorption of both PA and OA into PDMS after 24 h of incubation, majority of the FFAs were still absorbed. Therefore, the medium was replaced every 6 h during the experiments. However, further improvements are required to prevent PDMS absorption, and other structural materials should be used for OOCs. This has long been a topic of discussion in this research field^17,35^.

### Modeling GLA in the chip

**Fig. 2:**
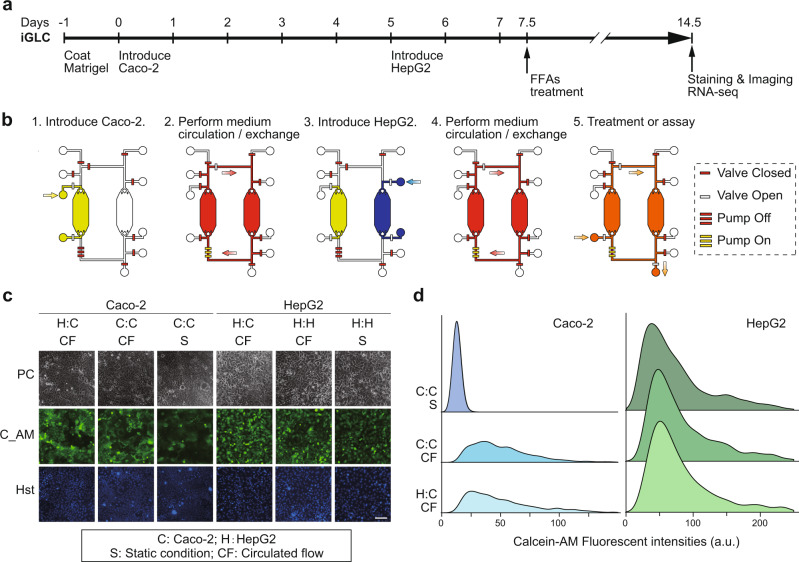
Establishment of the GLA in the iGLC platform. **a**, **b** Experimental procedure to culture Caco-2 and HepG2 cells in an iGLC platform (see also Supplementary Methods). Briefly, after a microfluidic channel was washed with a fresh cell culture medium, all valves were closed to prevent air contamination into the chip. Two valves next to the cell culture chamber were opened, and cell suspensions of Caco-2 and HepG2 cells were separately introduced into the corresponding chambers. After 1 day of incubation at 37 °C to settle down the cells, the pump was actuated to circulate the medium. The cell culture medium was changed every 6 h. **c** Phase-contrast (PC) and fluorescent micrographs of Caco-2 and HepG2 cells with closed medium circulation in the iGLC platform and stained with Calcein AM (C_AM) and Hoechst 33258 (Hst). For comparison, we also assessed individually cultured Caco-2 (C:C) and HepG2 (H:H) cells under circulation flow (CF) and static conditions (S). H:C represents co-cultured Caco-2 and HepG2 cells. The scale bar represents 100 µm. **d** Ridgeline plots showing single-cell profiling of Caco-2 and HepG2 cells stained with Calcein AM. For comparison, we also assessed individually cultured Caco-2 (C:C) and HepG2 (H:H) cells under circulation flow (CF) and static conditions (S). H:C represents co-cultured Caco-2 and HepG2 cells. The *p*-values were estimated with the Tukey–Kramer test and are presented in Supplementary Table S1 for the Caco-2 and Supplementary Table S2 for HepG2 cells.

To recapitulate GLA in vitro, Caco-2 and HepG2 cells were introduced into individual cell culture chambers in the iGLC platform without cross-contamination, but with a closed circulation flow (Fig. 2a). To confirm that the iGLC platform allows sustainable cell cultivation, Caco-2 and HepG2 cells were cultured with a medium circulation flow of 15 nL min^−1^ for 7 days and evaluated using a Calcein AM fluorescent cell viability indicator (Fig. 2b–d and Supplementary Data 1). Although Caco-2 cells generally take approximately 21 days to obtain functionality, perfusion conditions allow Caco-2 cells to express their functions within a shorter time, such as 7 days^36,37^. Thus, we chose a 7-day cultivation time to recapitulate GLA in vitro. For comparison, we also assessed separately cultured Caco-2 and HepG2 cells in cell culture chambers at a flow rate of 15 nL min^−1^ under static conditions. Based on computational fluid dynamics (CFD), 3.53 × 10^−4^ dyne cm^−2^ of fluid shear stress (FSS) was generated on the cells (Supplementary Fig. S6). The flow condition did not affect the viability of HepG2 cells, which showed minor improvement with a circulating flow. The Caco-2 cells showed increased viability with a circulating flow compared to that with static conditions. The fluorescence intensity of Calcein AM increased approximately 7.4-fold with the circulating flow. Caco-2 cells have previously been reported to show improved functionality and viability when cultured under continuous perfusion on a microfluidic device within 1-week of culture^36,37^. This proves that the iGLC platform provides better circulation flow conditions for both Caco-2 and HepG2 cells.

### Induction of NAFLD on a chip

**Fig. 3:**
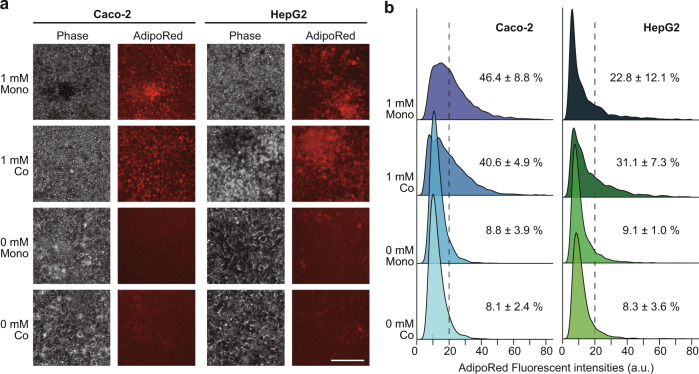
Lipids accumulated in both Caco-2 and HepG2 cells after 7-day FFAs treatment. **a** Phase contrast and fluorescent micrographs of Caco-2 and HepG2 cells treated with FFAs (0 and 1 mM) for 7 days stained with AdipoRed lipid fluorescent dye. The scale bars represent 100 µm. **b** Ridgeline plots to evaluate FFAs accumulation in individual cells [Caco-2 (*left*) and HepG2 (*right*)] after FFAs treatment for 7 days. The *p*-values were estimated with the Tukey–Kramer test and are presented in Supplementary Tables S5, S6 for the Caco-2 and HepG2 cells, respectively. Cells with over 20 of AdipoRed fluorescent intensity were considered as lipid accumulated cells. The data represent the mean ± standard deviation (*n* = 3).

**Fig. 4:**
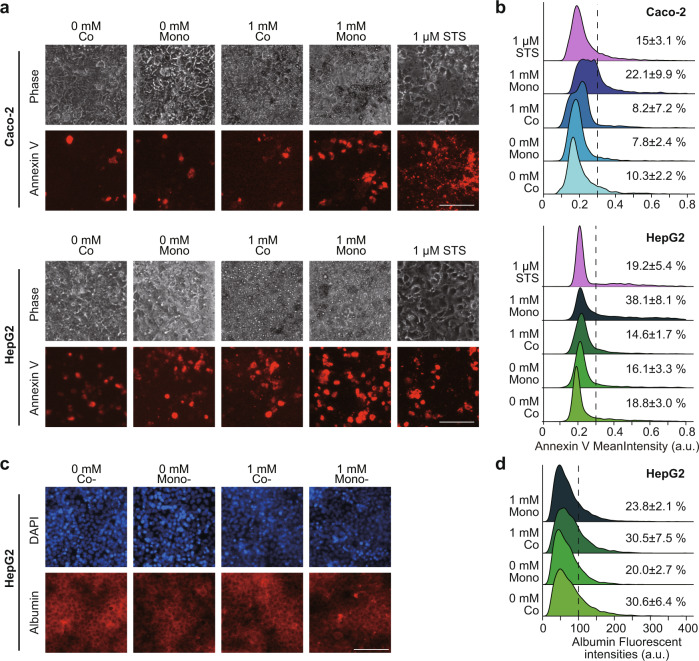
Accumulated lipids cause apoptosis in both Caco-2 and HepG2 cells. **a** Phase contrast and fluorescent micrographs of Caco-2 and HepG2 cells treated with FFAs (0 and 1 mM) stained with the Annexin V apoptotic cell marker. The scale bars represent 100 µm. **b** Ridgeline plots to evaluate individual apoptotic cells (Caco-2 and HepG2) after FFAs treatment for 7 days. For comparison, cells were treated with 1 µM of staurosporine (STS) for 24 h. The *p*-values were estimated with the Tukey–Kramer test and are presented in Supplementary Tables S7, S8 for the Caco-2 and HepG2 cells, respectively. Cells with over 0.3 of Annexin V fluorescent intensity were considered as apoptotic cells. **c** Phase contrast and fluorescent micrographs of HepG2 cells treated with FFAs (0 and 1 mM) stained with the human albumin. The scale bars represent 100 µm. **d** Ridgeline plots to evaluate individual HepG2 cells with human albumin expression after FFAs treatment for 7 days. The *p*-values were estimated with the Tukey–Kramer test and are presented in Supplementary Table S9 for the HepG2 cells. Cells with over 100 of ALB fluorescent intensity were considered as ALB-expressing cells. The data represent the mean ± standard deviation (*n* = 3).

To induce NAFLD in the iGLC platform, we used FFAs represented by a mixture of PA and OA, which is typical of Western diets (see Methods)^38^. As shown in Fig. 2a, prior to the FFAs treatment, Caco-2 and HepG2 cells were cultured in the iGLC platform with Dulbecco’s modified Eagle’s medium (DMEM) supplemented with 10%(v/v) FBS, and then, replaced to serum-free DMEM for 12 h to ensure cell starvation. A series of FFAs concentrations from 0 to 2 mM were introduced into the iGLC platform, and the cells were incubated for 24 h with medium circulation. To evaluate the FFAs accumulation in the mono-cultured Caco-2 and HepG2 cells, the cells were stained with AdipoRed lipid fluorescent dye to visualize the intracellular lipids (Supplementary Fig. S7 and Supplementary Tables S3 and S4). Both Caco-2 and HepG2 cells showed dose-dependent intracellular FFAs accumulation, and 1 mM FFAs treatment was used for further studies. Lipid droplet accumulation in hepatocytes is a hallmark of NAFLD^39^ and was observed in our model. During FFAs treatments, we used serum-free medium supplemented with 1% (w/v) BSA to minimize the effects of cell proliferation. Both mono-cultured Caco-2 and HepG2 cells showed a reduction in cell proliferation and did not interfere with cell treatment because of over-cell growth (Supplementary Fig. S8). We then examined two treatment periods, 1 and 7 days, representing NAFLD initiation and progression, respectively. We also confirmed that the FFAs treatment for 7 days increased intracellular lipid accumulation in mono- and co-cultured Caco-2 and HepG2 cells (Fig. 3a, b and Supplementary Data 2). The co-cultured Caco-2 cells showed less lipid accumulation than the mono-cultured cells, but HepG2 cells showed increased lipid accumulation. We also performed Annexin-V staining to evaluate apoptotic status after FFAs treatment. For comparison, the cells were also treated with 1 µM staurosporine (STS), which induces apoptosis. Although PA accumulation has been reported to cause cytotoxicity after 1-day of treatment^40,41^, the apoptotic staining (Fig. 4a) followed by quantitative single-cell profiling (Fig. 4b and Supplementary Data 3) results suggested that the 7-day FFAs treatment induced apoptosis in mono-cultured Caco-2 and HepG2 cells, whereas 1-day treatment did not induce apoptosis (Supplementary Fig. S9). However, the co-culture of Caco-2 and HepG2 cells showed a reduction in apoptosis. Notably, the expression of albumin (ALB), a functional hepatocyte marker, in mono-cultured HepG2 cells was not changed by FFAs treatment, but co-cultured HepG2 cells demonstrated increased ALB expression (Fig. 4c, d and Supplementary Data 3). This suggested that HepG2 cells co-cultured with Caco-2 cells displayed improved functionality.

**Fig. 5:**
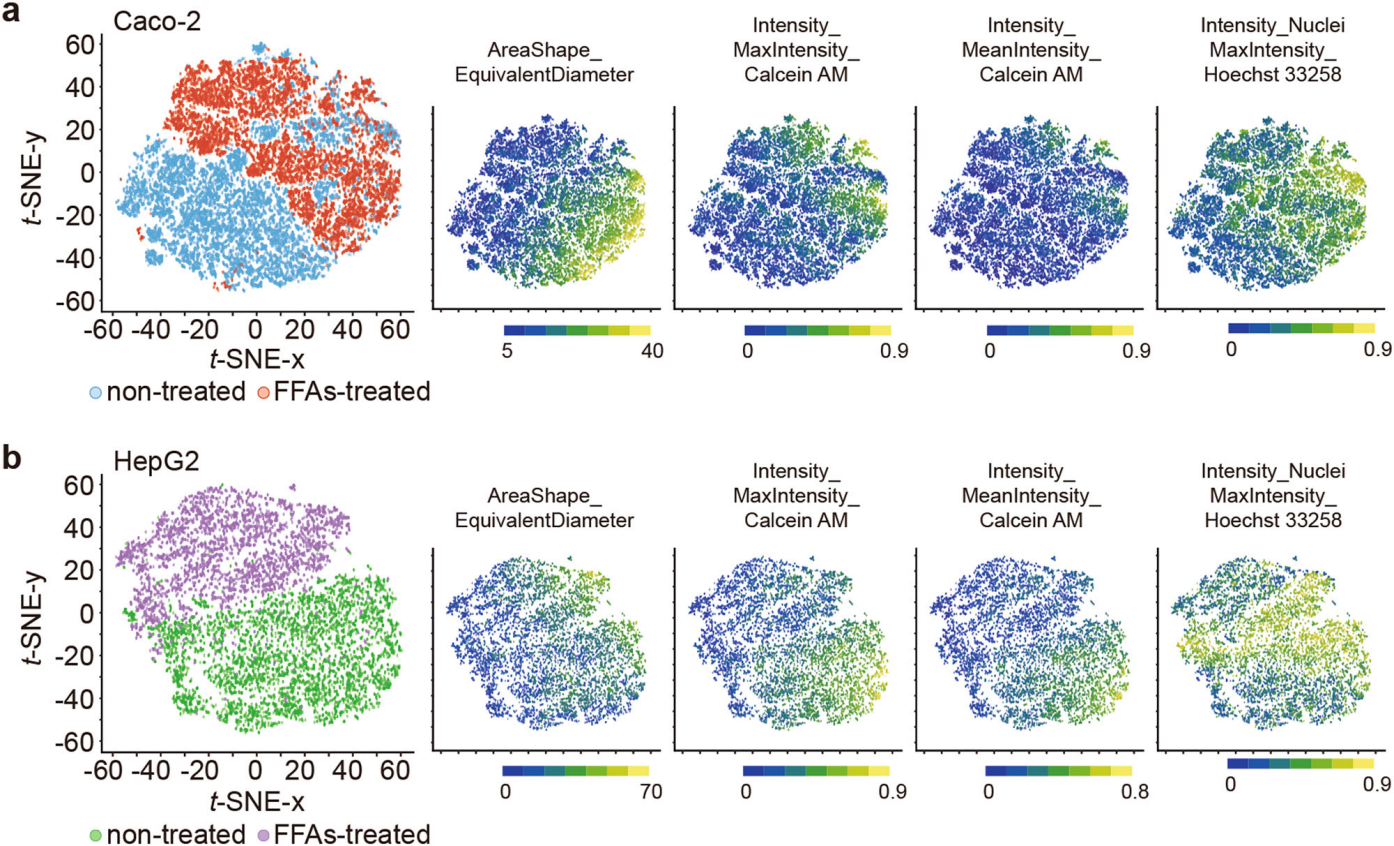
Single-cell profiling for the t-SNE analysis. **a**, **b** Two-dimensional t-SNE plots of microscopic single-cell profiling of co-cultured Caco-2 (**a**) and HepG2 (**b**) treated with 1 mM of FFAs or no treatment and stained with Calcein AM cellular and Hoechst 33258 nuclei markers (*n* = 3). The most distinguishable cellular parameters (AreaShape_EquivalentDiameter, Intensity_MaxIntensity_Calcein AM and Intensity_MaxIntensity_Hoechst 33258) are shown in the corresponding t-SNE plots, as well as the boxplots in Supplementary Fig. S11. The *p*-values are presented in Supplementary Tables S11,S12.

To further evaluate the cellular phenotypes after the FFAs treatment in detail, we stained cells with Calcein AM and Hoechst 33258 fluorescent dyes. This was followed by high-content analysis (HCA) using the CellProfiler computer-guided single-cell analysis software (Fig. 5, Supplementary Data 4, Supplementary Figs. S10, S11)^42,43^. In general, the Calcein AM and Hoechst 33258 dyes are used for cell viability function and as nuclear markers. Calcein AM staining can also be applied to evaluate cell and nucleus morphologies to obtain cellular parameters for HCA^44,45^. Moreover, recent advances in single-cell profiling based on high-content images, such as *t*-distributed stochastic neighbor embedding (t-SNE), have allowed a quantitative understanding of individual cellular status with high-dimensional cellular parameters and visualizing such parameters in a two-dimensional map^46^. We analyzed microscopic images to quantify 68 cellular parameters (Supplementary Data 4 and Supplementary Table S10) for each cell of Caco-2 and HepG2 cells treated with 1 mM FFAs for 7 days (Fig. 5a, b and Supplementary Data 4). We identified specific parameters that could be distinguished between non-treated and FFAs-treated cells that are associated with the cellular shape (AreaShape_FormFactor, AreaShape_MeanRadius, and AreaShape_EquivalentDiameter), Calcein AM intensity (Intensity_LowerQuartileIntensity, Intensity_MeanIntensity, and Intensity_MaxIntensity), nucleus shape (Nucleus_AreaShape_FormFactor), and Hoechst 33258 nuclei intensity (Intensity_LowerQuaterileIntensity, Intensity_MeanIntensity, Intensity_MaxIntensity, and Intensity_MinIntensityEdge). Notably, for the Caco-2 cells, the most distinguishing features were the cellular shape (AreaShape_EquivalentDiameter), Hoechst intensity (Intensity_MaxIntensity), and Calcein AM intensity (Intensity_MaxIntensity) (Fig. 5a). However, for the HepG2 cells, the most distinguishing feature was the Calcein AM intensity (Intensity_MeanIntensity and Intensity_MaxIntensity), the FFAs-treated HepG2 cells showed a reduction in these features (Fig. 5b). Thus, HCA based on simple cell and nucleus staining can be used to identify minute cellular phenotypic changes upon FFAs treatment that cannot be distinguished using molecular apoptotic markers. Because the iGLC platform does not require the use of cell culture inserts, it allows HCA-based single-cell profiling.

### mRNA-seq to identify gene expression signatures due to crosstalk for in vitro GLA treated with FFAs

**Fig. 6:**
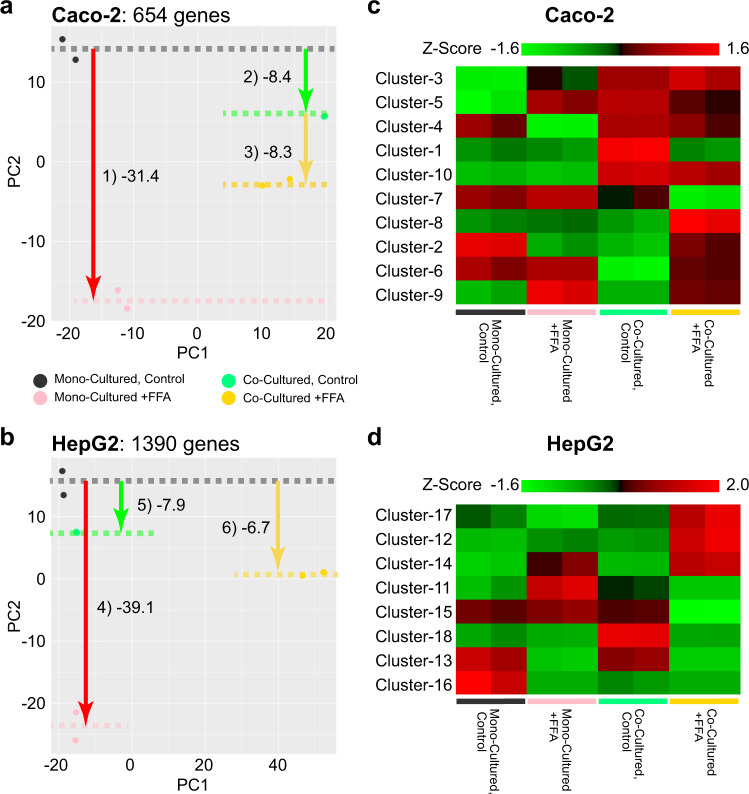
Gene expressions of the effects of FFAs treatment for 7 days and crosstalk with the in vitro human GLA model. **a**, **b** PCA results for DEGs obtained from the Caco-2 (**a**) and HepG2 (**b**) experimental sets. The mean PC2 values among the replicates are shown with dotted lines. For these diagrams, the gene name and change direction shown in (**a**, **b**) were considered. **c**, **d** Heat maps for the DEGs. *Z*-values of the expression profiles are shown.

To elucidate the effects of FFAs treatments and crosstalk via co-culturing on gene expression, we performed mRNA-seq, followed by PCA on the differentially expressed genes (DEGs), which were defined by an analysis of variance (ANOVA) (Fig. 6a, b, Supplementary Figs. S12, S13, Supplementary Data 5, 6 and Methods). We identified 654 and 1330 genes in Caco-2 and HepG2 cells, respectively, that were differentially expressed among the mono- and co-cultured conditions with or without 1 mM FFAs treatments for 7 days. In Caco-2 cells, the PC1 axis distinguished mono- and co-culture conditions, whereas the PC2 axis distinguished FFAs treatment. Notably, the co-culture conditions showed less of an effect on gene expression altered by FFAs treatments. In contrast, in the case of HepG2 cells, the PC1 axis clearly distinguished HepG2 cells treated with 1 mM FFAs under co-culture conditions from those treated with the other conditions. The PC2 axis in PCA of HepG2 cells also showed changes in gene expression associated with FFAs treatment; however, co-culturing reduced the changes in gene expression following FFAs treatment.

The heat map of K-means clustering for DEGs in Caco-2 cells showed multiple clusters with similar upregulated gene expression patterns under co-culture conditions, compared to mono-culture conditions (Clusters 1, 3, 5, and 10 in Fig. 6c). These clusters were related to cell cycle and metabolic processes, as suggested by gene ontology (GO) terms (Supplementary Figs. S14, S15). We also observed downregulated genes specific to the co-culture condition in Caco-2 cells (Clusters 2 and 6); however, the number of genes were too small to obtain sufficient interpretations from GO analysis. In the case of HepG2 cells, Cluster 18 in the heat map for DEGs showed unique upregulated genes in the co-culture condition, whereas only Cluster 16 showed downregulated genes specific to the co-culture condition (Fig. 6d, and Supplementary Figs. S16, S17).

**Fig. 7:**
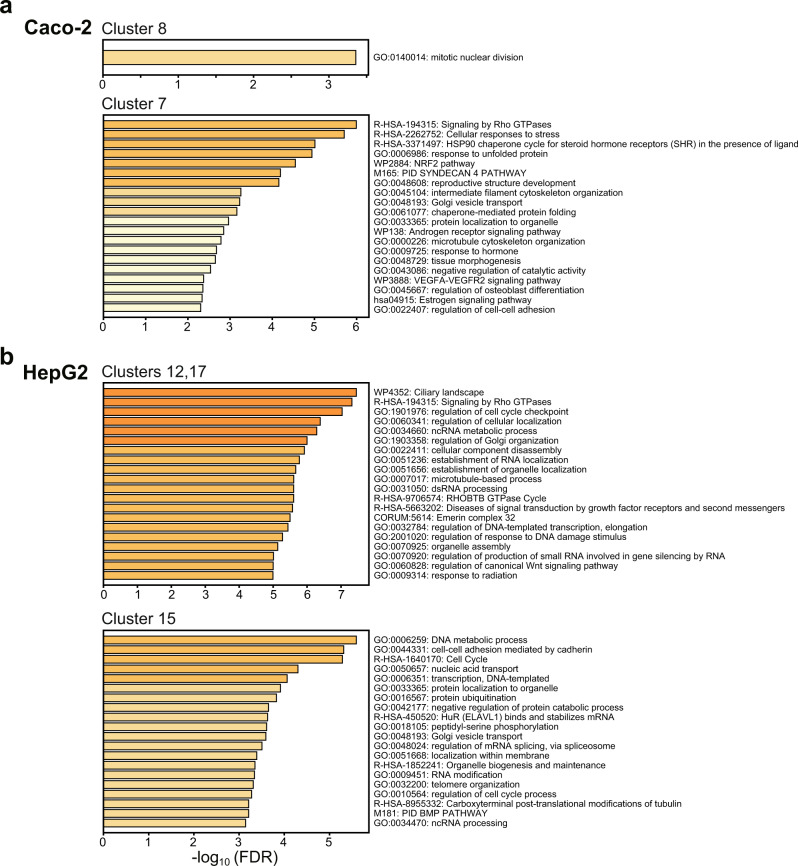
Enriched gene ontology terms of the FFAs-treated Caco-2 and HepG2 cells under mono- and co-cultured conditions. **a**, **b** Bar graphs showing gene enrichment related to certain GO terms and pathways for representative K-means clusters of FFAs-treated Caco-2 and HepG2 cells under mono- and co-cultured conditions. The other clusters were shown in Supplementary Figs. S14–S17.

We then compared the effects of FFAs treatment under mono- and coculture conditions on gene expression in Caco-2 and HepG2 cells. The mRNA-seq results revealed that Caco-2 cells showed elevated expression of genes associated with mitosis (Cluster 8 in Figs. 6a, 7a), whereas stress-responsive genes (Cluster 7 in Figs. 6a, 7a) were downregulated. In contrast, HepG2 cells treated with FFAs displayed elevated expression of genes related to the cell cycle (Clusters 12 and 17 in Fig. 7b)^47^, and suppression of the expression of genes associated with cell-cell adhesion and nucleic acid transport (Cluster 15 in Fig. 7b).

## Discussion

We established a human iGLC platform to investigate GLA response to FFAs in an NAFLD model. Although OOCs have been used to study NAFLD, the iGLC platform has multiple advantages owing to its application of microfabrication technology. Integrating the microvalves and a pump in the platform enables accessibility to individual cell culture chambers without undesired cross-contamination and closed medium circulation to mimic human GLA.

To investigate the effects of FSS on cells caused by closed-circulating flow, we conducted CFD to estimate the FSS generated by closed-circulation flow. The result was 3.53 × 10^−4^ dyne cm^−2^ (Supplementary Fig. S6). Under these flow conditions, Caco-2 cells showed increased cell viability (Fig. 2c), while HepG2 cells did not show any change (Fig. 2d). A number of previous reports have suggested that Caco-2 cell functionality can be improved by applying perfusion flow at over 0.0002 dyne cm^−2^, which is similar to the FSS level we tested^48–51^. Additionally, functional activation by FSS in HepG2 cells has been reported, but it requires a higher FSS of over 0.1 dyne cm^−2^ ^52,53^.

Notably, co-cultured HepG2 and Caco-2 cells significantly changed their gene expression profiles compared to mono-cultured cells (Clusters 1, 3, 5, 10, and 18 in Fig. 6c, d, and Supplementary Figs. S14–S17). The upregulated genes in Caco-2 cells were related to Mitotic G1 phase and G1/S transition, respiratory electron transport, ATP synthesis by chemiosmotic coupling, heat production by uncoupling proteins, positive regulation of spindle checkpoint, and small-molecule catabolic processes. Most of them were associated with the cell cycle and metabolic processes, but the cell proliferation analysis did not show a significant difference between co- and mono-cultured Caco-2 cells (Supplementary Fig. S8). Thus, gene expression in Caco-2 cells co-cultured with HepG2 cells did not affect the cell cycle and proliferation. In contrast, HepG2 cells upregulated genes showed GO terms of ‘plasma membrane-bound cell projection assembly (Cluster 18 in Supplementary Fig. S17), and downregulated genes showed ‘microtubule-based movement’ (Cluster 16 in Supplementary Fig. S17). Similarly, we did not observe any obvious cellular phenotypes associated with changes in gene expression.

To recapitulate NAFLD-like GLA in vitro, FFAs were administered to the cells in the iGLC platform (Fig. 2a). Although lipid accumulation occurred in both Caco-2 and HepG2 cells treated with FFAs for 7 days (Fig. 3a, b), the numbers of apoptotic Caco-2 and HepG2 cells in the co-culture condition were significantly reduced compared to those in mono-culture conditions.

In the case of Caco-2 cells, FFAs treatment for 1 day did not affect the apoptotic cellular status in either condition (Supplementary Fig. S9). Enriched GO terms are presented in Supplementary Fig. S13 is associated with cellular response to copper ions. Copper is an essential trace element for human physiological processes, including enzymatic metabolism^54^. Dysregulation of intracellular copper ions generates reactive oxygen species (ROS) and metabolic imbalance, which results in DNA damage and apoptosis^55–57^. Our results suggest that Caco-2 cells treated with FFAs for 1 day did not show an apoptotic cellular state with cellular membrane disruption, but initiated DNA damage due to the cellular response to copper ions. In contrast, even though the prolonged 7-day FFAs-treated mono-cultured Caco-2 cells showed an increase in the count of apoptotic cells (22.9 ± 9.9%), the 7-day FFAs-treated Caco-2 cells co-cultured with HepG2 cells had protective effects against lipid-induced apoptosis (8.2 ± 7.2%), and similar levels were observed in non-treated Caco-2 cells (mono-cultured, 7.8 ± 2.4%; co-cultured, 10.3 ± 2.2%). Previously, although intestinal cells treated with FFAs showed apoptosis due to lipid accumulation followed by an increase in the levels of reactive oxygen species^58,59^, our microscopic single-cell profiling suggests suppression of the apoptotic signaling, which is in agreement with mRNA-seq results shown in Figs. 6a, c and 7a, indicating the down-regulation of ‘Signaling by Rho GTPase,’ the typical apoptosis-associated signaling pathway^60^.

In addition to examining the apoptotic status of Caco-2 cells, we also further examined the correlation between phenotypic changes in cellular morphology and gene regulation networks for in vitro GLA in the NAFLD-like state by single-cell profiling (Fig. 5 and Supplementary Fig. S10). At the initiation stage of 1-day FFAs treatment, Hoechst 33258 intensity was slightly higher in non-treated Caco-2 cells than in FFAs-treated Caco-2 cells (Supplementary Figs. S10), but at the progression stage by 7-day FFAs treatment, the Hoechst 33258 intensity showed the opposite result, where FFAs-treated Caco-2 cells showed a higher intensity than the non-treated Caco-2 cells (Fig. 5). In general, cellular uptake of Hoechst 33258 can occur either by ABC transporters or by diffusion via a damaged cellular membrane. The mRNA-seq results for Caco-2 cells treated with FFAs for 1 or 7 days (Supplementary Figs. S13–S15) did not indicate any enriched GO terms associated with transporters (i.e., ATP-binding cassette transporter ABCG2)^61^ that may efflux Hoechst 33258 outside the cells. Thus, the transporters did not affect the Hoechst 33258 intensity. Therefore, FFAs-treatment might damage the cellular membrane, resulting in an increase in Hoechst 33258 intensity, but not induction of apoptosis.

In contrast, FFAs-treated HepG2 cells had the most distinguishable features, such as Calcein AM intensity (Intensity_MeanIntensity and Intensity_MaxIntensity), and FFAs-treated HepG2 cells showed a reduction in these features (Fig. 5b). Considering that the intensity of Calcein AM represents cell viability^62^, this result suggests that FFAs reduce the viability of co-cultured HepG2 cells. In contrast, we did not observe an increase in apoptotic HepG2 cells under co-culture conditions, as confirmed by Annexin V staining (Fig. 4a, b). Notably, the mRNA-seq results revealed that HepG2 cells co-cultured with Caco-2 cells showed increased gene expression associated with the cell cycle (Clusters 12 and 17 in Fig. 7b). In addition, the expression of genes associated with cell-cell adhesion was reduced in FFAs-treated HepG2 cells under co-culture conditions (Cluster 15 in Fig. 7b). These results suggested that, as FFAs treatment induced mild damage in HepG2 cells co-cultured with Caco-2 cells, HepG2 cells transformed from differentiated liver cells to proliferating cells. Cell cycles and cell division are essential to maintain tissue homeostasis, but in diseases such as NAFLD, they often highlight the imbalance of the cell cycle caused by disrupted metabolic signaling^47^. Overall, the results with our platform indicate that HepG2 cells co-cultured with Caco-2 cells were activated towards NAFLD-like gene expression networks with FFAs treatment.

To develop an advanced in vitro NAFLD model, the cell types used are critical. In this study, HepG2 human hepatocellular carcinoma cell line and Caco-2 human colorectal adenocarcinoma cell line were used since they have been immortalized and widely used until now. However, such cell lines have genetic mutations and cannot express proper functions as an actual hepatocyte^63^ and intestinal cells^64^. In the case of hepatocytes, the HepaRG human hepatocellular carcinoma cell line has been used for liver disease modeling as well as toxicological research due to their relatively higher functionalities than those of HepG2 cells recently, but HepaRG cells also showed differences in lipid metabolism^65^. Moreover, cell lines generally form a pure cellular population, and cannot represent the cellular diversity. For example, the liver contains hepatocytes and non-parenchymal cells (NPCs) such as Kupffer cells, endothelial cells, and stellate cells. In particular, since the liver microphysiological system (L-MPSs) was reported to recapitulate liver steatosis using NPCs^66^, NPCs are now recognized for their importance in hepatic disease modeling. In contrast, primary cells obtained from NAFLD patients showed similar cellular diversity and drug metabolic responses^67^. However, primary cells have limited cell growth capability and often lose their functions in vitro. Moreover, it is difficult to identify healthy donors to obtain a control sample. In addition to primary cells, human pluripotent stem cells (hPSCs), such as embryonic^68^ and induced pluripotent stem cells^69–71^, can provide multiple cell types from a single-cell source after induction of differentiation by applying growth factors and nutrients. The recent advancement of gene editing technologies, such as CRISPR/Cas systems^72^, allows the creation of gene-modified hPSCs used for disease modeling, for which the primary cells are not applicable. However, the tissue cells derived from hPSCs could not fully cover the entire cellular diversity nor express their functionality in vivo. Therefore, it remains challenging for hPSCs to fully recapitulate the pathological conditions of NAFLD in vitro. In this study, although hepatic and intestinal cell lines were used to recapitulate GLA and NALFD, they were able to provide new insights into NAFLD-like gene expression networks upon FFAs treatment. Using primary cells or hPSC-derived hepatocytes/intestinal cells with higher functionality and cellular diversity, the iGLC platform will allow the reproduction of the ideal results of in vivo NAFLD initiation and progression.

Moreover, as demonstrated in this study, NAFLD is involved in organ-to-organ interactions. Recent reports have suggested that the gut microbiota significantly impacts NAFLD development and needs to be considered for establishing a new NAFLD model^9–11^. However, each organ often requires optimal culture conditions to express its proper functions. Our current iGLC platform can only use the same cell-culture medium to interconnect two different tissues; therefore, the next generation of iGLC platforms needs to overcome these issues. Recently, we reported the multilayered GLA-MPS^27,73^ to realize both individual accessibility for each tissue and closed loop medium circulation, for recapitulating inflammatory bowel disease by treating intestinal cells with an inflammatory inducer (e.g., lipopolysaccharide). However, the multilayered GLA-MPS device was made of PDMS and was not suitable for modeling NAFLD without any surface treatment against molecular absorption, such as FFAs^28^. As shown in Supplementary Fig. S5, because the surface coating for PDMS reduced FFAs absorption, and the multilayered GLA-MPS with the PDMS surface coating will be able to serve as a platform to study NAFLD initiation and progression by treating cells with FFAs in a more quantitative and robust fashion.

Instead, it is also valuable to find alternative materials to PDMS to avoid the well-known concerns of PDMS^74,75^. Thermoplastic materials (e.g., polystyrene, polymethyl methacrylate, polypropylene, and cyclo-olefin polymers) could be applicable for fabricating OOCs and mass production, but it is difficult to deform membranes for actuating microvalves/pumps or stretching cultured cells^76,77^, owing to their rigidity. Perfluoropolyether elastomers have recently been used for fabricating OOCs enabling the prevention of the absorption of hydrophobic molecules^78,79^. Although perfluoropolyether elastomers could be attractive for modeling NAFLD in vitro, such elastic materials still pose a challenge for mass production for industrial applications. Therefore, it is critical to choose the optimal materials for fabricating OOCs to fulfil the requirements of the targeted studies and applications to obtain more accurate results representing human pathophysiological conditions^80^.

In summary, the iGLC platform represents a new in vitro human model for recapitulating physiological and NAFLD pathological conditions, with a focus on GLA. In the near future, the iGLC platform combined with HCA and omics approach may provide deeper insights into NAFLD development for the establishment of new drugs. The iGLC platform can contribute to the development of drugs for NAFLD, as well as a variety of disorders associated with GLA^81^, such as inflammatory bowel disease, which do not have any in vitro experimental settings.

## Methods

### Chip fabrication

The iGLC platform was fabricated from a flexible PDMS (SYLGARD 184, Dow Corning) polymer using a multilayer soft lithography replica molding technique, as previously reported (Supplementary Fig. S1)^82,83^. Briefly, the control layer consisted of microchannels supplying hydraulic pressure that were cast from a 30 µm thickness of negative photoresist mold (TMMR S2000, Tokyo Ohka Kogyo) and patterned using a standard photolithography tool. The perfusion layer consists of two cell culture chambers (225 µm in height and 2.1 mm in width) interconnected by semi-elliptical microchannels (45 µm in height and 200 µm in width). The mold fabrication process for the perfusion layer followed the multilayer lithography principle, which combines standard photolithography (cell culture chambers) and grayscale lithography (microchannels). First, a negative resist layer (TMMF S2045, Tokyo Ohka Kogyo) with a thickness of 180 µm was patterned on a silicon wafer. Next, a positive resist (PMER P-LA900PM, Tokyo Ohka Kogyo) was spin-coated with a thickness of 45 µm on the wafer. Subsequently, digital micromirror device (DMD)-based grayscale lithography (DL-1000GS/KCH, NanoSystem Solutions) was performed using numerically optimized mask data^82^ to achieve precise wafer-level mold fabrication. This allowed complete sealing of the microchannels with microvalves and high-efficiency driving of the peristaltic micropumping system. After the mold fabrication, the PDMS base and curing agent were thoroughly mixed at a weight ratio of 10:1. For the control layer, the PDMS mixture was spin-coated with a thickness of 50 µm on the mold to obtain a controlled PDMS thickness of 20 µm at the membrane portion. The PDMS for the control and perfusion layers was cured on a hotplate at 80 °C for 4 min and in a convection oven at 80 °C for 40 min, respectively. The perfusion layer was then peeled off, precisely aligned, and bonded to the control layer using a partial PDMS curing method. The assembled structure was placed in an oven at 80 °C for 2 h and then peeled from the silicon wafer. Finally, the inlet and outlet wells were opened. The assembled device was permanently bonded using O_2_ plasma (FA-1, SAMCO) onto a microscopic slide glass (25 mm × 75 mm).

### Device control

The microvalves and micropump were actuated by positive hydraulic pressure via linked control channels. The control channel within the chip was first filled with distilled water using a 1 mL syringe to prevent gas permeation across the PDMS. Metal pins and Teflon tubes (Pilot Corporation) were then used to connect the inlet wells of the control channels and pneumatic system with a compressed nitrogen gas resource (regulated at 0–200 kPa). The pressure actuation and release of the valves were controlled and operated with LabVIEW software (Version 11.0, National Instruments) via solenoid valves (Microfluidic System Works Inc. and LEE Company) using a controller board (VC3 8 controller [ALA Scientific Instruments] and NI USB-6501 [National Instruments]). The micropump consists of three adjacent microvalves with sequential actuation to provide periodic peristaltic motion to generate a medium recirculation flow in the chip.

### Cell culturing

HepG2 human hepatocellular carcinoma and Caco-2 human colorectal adenocarcinoma cell lines were obtained from the American Type Culture Collection. Cells were maintained in Dulbecco’s modified Eagle’s medium (DMEM) (Sigma-Aldrich, St. Louis, MO, USA) supplemented with 10% (v/v) fetal bovine serum (FBS, Cell Culture Bioscience), 1% (v/v) nonessential amino acids (Thermo Fisher Scientific), and 1% (v/v) penicillin/streptomycin (Thermo Fisher Scientific) in a humidified incubator at 37 °C with 5% (v/v) CO_2_. HepG2 and Caco-2 cells were passaged using trypsin/EDTA (0.04% / 0.03%[v/v]) solution every 3 and 5 days and at ratios of 1:5 and 1:10, respectively.

To ensure the absence of mycoplasma contamination in cellular samples, a mycoplasma detection assay was conducted by using the MycoAlert™ mycoplasma detection kit (Lonza, LT07-118) according to the manufacturer’s instructions. The positive and negative controls were included in each assay run to ensure the accuracy of the results. By measuring the luminescence before (read A) and after the addition of the MycoAlert™ Substrate (read B), a ratio was used to identify the presence or absence of mycoplasma. The HepG2 and Caco-2 cells have 0.7 and 0.3 B/A ratio, respectively. Then, compared with negative control 0.4 and positive control 18.6, the cellular samples (<0.9) were confirmed no mycoplasma contamination.

### Cell culturing on a platform

Before cell seeding, the platform was sterilized by washing with 70% ethanol and placed under ultraviolet light in a biosafety cabinet for 30 min. Subsequently, the cell culture chambers were coated with 0.1% (w/v) DDM (*n*-dodecyl β-D-maltoside) in PBS at 4 °C for 24 h and then coated with Matrigel hESC-qualified matrix (Corning) diluted to 1.3% (v/v) in DMEM/F12 (Sigma-Aldrich) at 4 °C for 24 h (Supplementary Fig. S3). After the excess Matrigel was rinsed with DMEM, the chip was placed in an incubator at 37 °C until use.

HepG2 and Caco-2 cells were harvested from the culture flasks with 1 mL of trypsin/EDTA (0.04%/0.03% [v/v]) solution and incubated at 37 °C for 5 min. After centrifugation, the cells were resuspended in fresh cell culture medium at 1.0 × 10^6^ cells mL^−1^. The microvalves next to the cell culture chambers were closed to prevent cross-contamination during cell seeding. Subsequently, 5 µL of the Caco-2 cell suspension was introduced with a pipette into the well adjacent to the cell culture chamber at 7.0 × 10^4^ cells cm^−2^. HepG2 cell suspension (5 µL) was introduced into another cell culture chamber. The platform was placed in a humidified incubator at 37 °C and 5% (v/v) CO_2_. After one day, the cell culture medium was changed to remove floating dead cells. The cell culture medium was changed every 6 h under the control of our custom LabVIEW-based software.

### Free fatty acid treatment

The FFAs treatment solutions were a mixture of PA (Sigma-Aldrich) and OA (Sigma-Aldrich) at a molar ratio of 1:2. To prepare the treatment solutions, PA was dissolved in dimethyl sulfoxide (DMSO; Nacalai Tesque Inc.) at 20 mg mL^−1^ to serve as the PA stock solution (78 mM). OA was dissolved in DMSO at 100 mg mL^−1^ to serve as the OA stock solution (354 mM). PA and OA stock solutions were mixed (PA:OA = 1:2) in DMEM (containing 1% BSA fatty acid-free, 1% P/S, and 1 mM nonessential amino acids) to generate a series of FFAs concentrations (0.1, 0.2, 0.5, 1, and 2 mM). Prior to FFAs treatment, both the cell culture chambers were replaced with serum-free DMEM for 12 h of cell starvation. FFAs-containing medium was then introduced into the cell culture chamber. The platform was incubated at 37 °C in a humidified incubator for 7 days, and the medium was exchanged every 6 h.

### Cell viability and apoptosis staining

Calcein AM (Dojindo Molecular Technologies, Inc.) and Annexin V-Alexa Fluor® 647 (BioLegend) dyes were used to stain viable and apoptotic cells, respectively. Hoechst 33258 (Dojindo Molecular Technologies, Inc.) was used to stain the nuclei. The staining solution was prepared by mixing 10 µL Hoechst 33258 (1 mg mL^−1^ stock concentration), 10 µL Calcein AM (1 mg mL^−1^ stock concentration), 10 µL Annexin V (50 µg mL^−1^ stock concentration), 500 µL Annexin V binding buffer (BioLegend), and 500 µL DMEM. The wash buffer comprised 500 µL of Annexin V binding buffer and 500 µL of DMEM. After treatment, the cells were washed twice with fresh DMEM, and 10 µL of staining solution was introduced into a cell culture chamber using a pipette via the adjacent inlet. The cells were then incubated at 37 °C for 30 min. Excess staining solution was removed by washing with 30 µL of wash buffer three times.

### AdipoRed staining

Lipid accumulation was visualized using the AdipoRed assay (Lonza), following the manufacturer’s protocol. Briefly, 15 µL of AdipoRed assay reagent and 10 µL of Hoechst 33258 were mixed with 1 mL of DMEM to prepare AdipoRed staining solution. The cell culture chambers and channels were washed twice with PBS. AdipoRed staining solution (10 µL) was added and incubated at 37 °C for 15 min. The chambers were washed three times with fresh DMEM solution.

### Immunocytochemistry

The cells in the cell culture chambers were washed with PBS. The cells were then fixed for 15 min with 4% paraformaldehyde (PFA, FUJIFILM Wako Pure Chemical) in PBS and permeabilized for 30 min with 0.1% (v/v) Triton X-100 in PBS. The cells were then incubated in blocking buffer containing 5% normal goat serum (Vector), 5% normal donkey serum (Wako), 3% BSA (Sigma-Aldrich), and 0.1% Tween-20 (Nacalai Tesque, Inc.) in PBS at 4 °C for 24 h. After blocking, the cells were treated with mouse anti-human albumin IgG (Stock solution 10 µg mL^−1^, diluted by blocking buffer into 20 ng mL^−1^, Thermo Fisher Scientific) as the primary antibody in blocking solution for 24 h. The following day, after washing the excess antibodies three times with PBS (containing 0.1% Tween-20), the cells were treated with Alexa Fluor 647-labeled donkey anti-mouse IgG H&L (Stock solution 1 µg mL^−1^, diluted by 0.1% Tween-20 PBS into 1 ng mL^−1^, Jackson ImmunoResearch) at 25 °C for 1 h. The nuclei of the cells were stained with 50 µL of a solution of 300 nM 4,6-diamidino-2-phenylindole (DAPI, Dojindo Laboratories) at 25 °C for 30 min and then washed twice with PBS.

### Image acquisition

The chips were placed on the stage of a Nikon ECLIPSE Ti inverted fluorescence microscope, which was equipped with a CFI plan fluor 10×/0.30 N.A. objective lens (Nikon, Tokyo, Japan), charge-coupled device (CCD) camera (ORCA-R2; Hamamatsu Photonics, Hamamatsu City, Japan), mercury lamp (Intensilight; Nikon), XYZ automated stage (Ti-S-ER motorized stage with encoders; Nikon), and filter cubes for the fluorescence channels (DAPI and GFP HYQ; Nikon). For image acquisition, the exposure times were set to 100 ms for (DAPI) Hoechst 33258, 5 ms for (GFP HYQ) Calcein AM, 2 s for (TRITC) Annexin V, and 100 ms for the (TRITC) AdipoRed assay.

### Ultrahigh-performance liquid chromatography-tandem mass spectrometry (UHPLC-MS/MS)

Firstly, 3 µL of the cell culture medium was collected from a chip and mixed with 96.95 µL of the working solution (50% isopropanol, 25% acetonitrile, and 25% water) and 0.05 µL of the internal standard solution (PA-d4:5 mM, OA-d9:5 mM, in isopropanol). The mixture was vigorously mixed for 30 s and centrifuged at 16,000 × *g* for 10 min at 4 °C. Subsequently, 2 µL of the supernatant was injected and separated on a Nexera UHPLC system (Shimadzu, Kyoto Japan) using a binary gradient with solvent A (50% acetonitrile, 18% isopropanol, and 32% water) and solvent B (90% isopropanol, 10% acetonitrile, 10 mM ammonium formate, and 0.1% formic acid). The gradient program was as follows: 0% B for 14 min, 100% B for 3 min, and 0% B for 3 min. An ACQUITY UPLC BEH C18 column (130 Å, 1.7 µm, 2.1 mm × 100 mm (Waters, Milford, MA)) was used at 40 °C. The UHPLC eluates were infused online to the LC-MS 8030plus (Shimadzu), which was set to negative electrospray ionization (ESI-) mode. The PA and OA responses were observed by pseudo multiple reaction monitoring (pMRM) with transitions at m/z 255.05 > 255.35 and 281.05 > 281.45, respectively. The pMRM transitions for PA-d4 and OA-d9 were 258.95 > 259.45 and 290.10 > 290.40, respectively. The pMRM transitions were optimized, and peak areas were calculated using LabSolutions software (Shimadzu). The PA and OA responses were normalized to those of PA-d4 and OA-d9 for each sample. All measurements were obtained in triplicate, and the average responses were used. Standard curves were generated by measuring blank culture medium supplemented with increasing amounts of PA and OA.

### RNA purification

RNA was purified from the cells using the RNeasy Mini Kit (Qiagen, Hilden, Germany). The microvalves were initially closed to prevent cross-contamination between the HepG2 and Caco-2 cells. After the cells were washed with PBS, 10 µL of trypsin/EDTA (0.04% / 0.03%[v/v]) solution was introduced into the cell culture chambers and incubated at 37 °C with 5% CO_2_ for 10 min. Subsequently, the cells were harvested with a 10-µL pipette and placed in 1.5-mL tubes. Cells were lysed by adding 350 µL of lysis buffer from the kit and 350 µL of 70% (v/v) ethanol to the tubes. Each solution was transferred to an RNeasy Mini spin column placed in a 2-mL collection tube. The column was centrifuged for 15 s at 8000 × *g* and the flow-through was discarded. Next, 350 µL buffer RW1 was added to the columns and centrifuged. Subsequently, 80 µL DNase digestion buffer was added to the column and incubated at 25 °C for 15 min. Subsequently, 350 µL of buffer RW1 was added to the column tube and centrifuged again. The column was washed twice with 500 µL of buffer RPE, placed in a new 2 mL tube, and centrifuged. The column was then placed in a new 1.5-mL collection tube and 30 µL of RNase-free water was added to the column. This was followed by centrifugation for 1 min at 8000 × *g* to elute RNA into the collection tube. RNA quality was evaluated using an Agilent 2100 Bioanalyzer (Agilent Technologies Inc., USA).

### RNA amplification and nanopore mRNA sequencing

mRNA-seq was conducted by Takara Bio Inc. or Oxford NANOPORE Technologies. For Takara Bio Inc, briefly, 50 ng of total RNA from each sample was amplified and synthesized to cDNA using SMART-seq (SMART-Seq v4 Ultra Low Input RNA Kit, Takara Bio). The cDNA was used to make a library using the Nextera DNA Flex Library Prep Kit (Illumina), and the cDNA library was sequenced using NovaSeq 6000 (Illumina). For Oxford NANOPORE Technologies, 40 ng of total RNA was diluted with 9 μL of RNase-free water, mixed with VN primer (Oxford NANOPORE Technologies, UK), and 1 μL of 10 mM dNTPs (New England Biolabs Inc. Ipswich, Massachusetts, USA), and incubated at 65 °C for 5 min to prepare the cDNA library. Separately, 4 μL of 5x RT Buffer (Thermo Fisher Scientific), 1 μL of RNaseOUT (Thermo Fisher Scientific), 1 μL of nuclease-free water, and 2 μL of Strand-Switching Primer (Oxford NANOPORE Technologies) were mixed as the strand-switching buffer. The two solutions were mixed at 42 °C for 2 min, and then 1 μL of Maxima H Minus Reverse Transcriptase (Thermo Fisher Scientific) was added. The mixture was incubated at 42 °C for 90 min and 85 °C for 5 min and stored at 4 °C until use as the cDNA library. Exactly 5 μL aliquot of the cDNA library solution was mixed with 25 μL of 2x LongAmp Taq Master Mix (New England Biolabs Inc.), 1.5 μL of Barcode Primers (Oxford NANOPORE Technologies), and 18.5 μL of nuclease-free water. PCR was performed (95 °C for 30 s; 18 cycles of 95 °C for 15 s, 62 °C for 15 s, 65 °C for 50 s, and 65 °C for 6 min) to barcode the cDNA for multiplexing. The PCR products were stored at 4 °C until use. Subsequently, 1 μL of NEB Exonuclease 1 (New England Biolabs Inc.) was added before incubation at 37 °C for 15 min, followed by incubation at 80 °C for 15 min. Amplified DNA was purified and collected in 12 μL of elution buffer (Oxford NANOPORE Technologies) using Agencourt AMPure XP beads (Beckman Coulter Life Sciences, Indianapolis, IN, USA). A BioAnalyzer 2100 with a High Sensitivity DNA Kit (Agilent Technologies) was used to evaluate the amount and quality of barcoded cDNA. Next, 50 fmol of barcoded cDNA was incubated with 1 μl of Rapid Adapter to make up a total volume of 11 μL, and this was incubated for 5 min at 25 °C. For Nanopore sequencing, 12 μL of the prepared DNA library was mixed with 37.5 μL of Sequencing Buffer (Oxford NANOPORE Technologies) and 25.5 μL of Loading Buffer (Oxford NANOPORE Technologies). This solution was added to a Nanopore Flow Cell (v9.4.1) and a sequencing run was performed for 24 h.

### mRNA-seq analysis

Initially, mRNA-seq reads were mapped to the rRNA, tRNA, or mitochondrial genome sequences using BowTie (v.2.1.0)^84^. The mapped reads were discarded and not used for the following analysis. The remaining reads were mapped to the human genome (GRCh38) with STAR Aligner (2.7.1a)^85^ using ENCODE options considering gene annotation and Ensembl (ver.98)^86^. After the mapping to the genome, gene expression values (Transcripts Per Million reads; TPM) were calculated using RSEM (ver. 1.3.0)^87^. DEGs of the mono-cultured and co-cultured samples were calculated using DEseq2 (ver. 1.8.2)^88^. If a gene satisfied the following criteria, it was defined as DEG: *p* < 0.01, abs (log_2_(Fold Change)) ≥ 0.263, base mean of raw reads ≥ 31, and average TPM in either sample ≥ 1. GO analysis of DEGs was performed using the WEB-based Gene Set Analysis Toolkit (WebGestalt^89^). ‘Biological Process noRedundant’ was selected for the database, and ‘genome protein-coding’ genes were selected for the reference set. Protein-coding genes among the DEGs were used as the inputs.

To consider FFAs treatment conditions, we employed ANOVA to select DEGs and characterize the samples. A gene was treated as a DEG if *p* < 0.05 and abs(log_2_(Fold Change)) ≥ 0.263 for a combination of any two samples. The expression values of the DEG were used for PCA to characterize the samples. To compare DEG sets with FFAs-minus and FFAs-plus under mono-culture conditions, FFAs-minus and FFAs-plus under the co-culture conditions, and mono- and co-cultured samples under the FFAs-minus condition, DEGs with a PC2 loading of ≥2 or ≤0.5 were used. The results were used to assess changes in gene expression related to FFAs treatment. To compare gene expression profiles according to FFAs treatment under the mono- and co-cultured conditions, we employed Gene Set Enrichment Analysis (GSEA^90^, ver.4.0.4) with DB files (msigdb.v7.1. symbols.gmt). The fold-change values for protein-coding genes were used as inputs. The bar plots show FDR qvalues for the top four terms under both mono- and co-culture conditions. In the analysis, the PCA plots were drawn with the ggplot2 package in R.

### Single-cell profiling based on microscopic images

Following microscopic image acquisition, CellProfiler software (Broad Institute of Harvard and MIT, Version 3.1.9)^43^ was used to estimate cellular features (e.g., cell size and fluorescence intensity). After loading the set of stained images (nuclei and cellular phenotype or function), all images were adjusted with “correctilluminationCalcuate” and “correctilluminationApply” modules to reduce the uneven distribution of background signals. The settings provided in the manual were followed. Traditionally, the illumination function is calculated from “Background” with a block size of 20–100 for each image individually. And the Smoothing method was selected as “Median filter,” with a filter size of 50. The other options followed the default settings. The mathematical methods of “correctilluminationApply” was “Substract.” With the corrected nuclei images, individual cells were identified by using Otsu’s method^43^ in the “IdentifyPrimaryObjects” module as primary objects, followed by “IdentifySecondaryObjects” to evaluate the cellular phenotype/function, automatically. Then, cellular features were calculated using the “MeasureObjectSizeShape” and “MeasureObjectIntensity” modules for both primary and secondary objects. Further analysis of single-cell morphological descriptors was performed using t-SNE in the open-source Orange 3 software (Version 3.23.1; Bioinformatics Laboratory, Faculty of Computer and Information Science, University of Ljubljana, Slovenia)^91^.

### Statistics and reproducibility

The results expression, statistical tests used, the sample sizes and number of independent experiments are mentioned in the figure legends. All cell-based assays were carried out in triplicate samples with three independent experiments. The Ridgeline plots and t-SNE analysis charts were based on the integration of three independent experiments. The Tukey–Kramer test and Student’s *t* test were performed using *R* software (ver. 3.5.2; https://www.r-project.org/). For mRNA-seq, all the experiments were conducted with at least two independent experiments.

### Reporting summary

Further information on research design is available in the Nature Portfolio Reporting Summary linked to this article.

## Supplementary information

Supplementary Information

Description of Additional Supplementary Files

Supplementary Data 1

Supplementary Data 2

Supplementary Data 3

Supplementary Data 4

Supplementary Data 5

Supplementary Data 6

Reporting Summary

## Data Availability

The mRNA-seq data has been deposited in the NCBI Gene Expression Omnibus (GEO) under accession number GSE152091 and GSE206417. All the manuscript graphs and charts of Ridgeline plots and t-SNE analysis are available in Supplementary Data 1 to 6.

## References

[1] Chalasani N (2018). The diagnosis and management of nonalcoholic fatty liver disease: Practice guidance from the American Association for the Study of Liver Diseases. Hepatology.

[2] Younossi ZM (2019). Non-alcoholic fatty liver disease—a global public health perspective. J. Hepatol..

[3] Byrne CD, Targher G (2015). NAFLD: A multisystem disease. J. Hepatol..

[4] Friedman SL, Neuschwander-Tetri BA, Rinella M, Sanyal AJ (2018). Mechanisms of NAFLD development and therapeutic strategies. Nat. Med..

[5] Estes C, Razavi H, Loomba R, Younossi Z, Sanyal AJ (2018). Modeling the epidemic of nonalcoholic fatty liver disease demonstrates an exponential increase in burden of disease. Hepatology.

[6] Buzzetti E, Pinzani M, Tsochatzis EA (2016). The multiple-hit pathogenesis of non-alcoholic fatty liver disease (NAFLD). Metabolism.

[7] Wiest R, Albillos A, Trauner M, Bajaj JS, Jalan R (2017). Targeting the gut-liver axis in liver disease. J. Hepatol..

[8] Sumida Y, Yoneda M (2018). Current and future pharmacological therapies for NAFLD/NASH. J. Gastroenterol.

[9] Ohtani N, Kawada N (2019). Role of the gut–liver axis in liver inflammation, fibrosis, and cancer: a special focus on the gut microbiota relationship. Hepatol. Commun..

[10] Leung C, Rivera L, Furness JB, Angus PW (2016). The role of the gut microbiota in NAFLD. Nat. Rev. Gastroenterol. Hepatol..

[11] Marchesi JR (2016). The gut microbiota and host health: a new clinical frontier. Gut.

[12] Clemente MG, Mandato C, Poeta M, Vajro P (2016). Pediatric non-alcoholic fatty liver disease: Recent solutions, unresolved issues, and future research directions. World J. Gastroenterol..

[13] Rotman Y, Sanyal AJ (2017). Current and upcoming pharmacotherapy for non-alcoholic fatty liver disease. Gut.

[14] Abdalkader R, Kamei KI (2020). Multi-corneal barrier-on-a-chip to recapitulate eye blinking shear stress forces. Lab Chip.

[15] Sung JH, Wang YI, Kim JH, Lee JM, Shuler ML (2018). Application of chemical reaction engineering principles to “body-on-a-chip” systems. AIChE J..

[16] Abaci HE, Shuler ML (2015). Human-on-a-chip design strategies and principles for physiologically based pharmacokinetics/pharmacodynamics modeling. Integr. Biol..

[17] Ronaldson-Bouchard K, Vunjak-Novakovic G (2018). Organs-on-a-chip: a fast track for engineered human tissues in drug development. Cell Stem Cell.

[18] Schurdak M (2020). Applications of the microphysiology systems database for experimental ADME-Tox and disease models. Lab Chip.

[19] Grego S (2017). Systems biology for organotypic cell cultures. ALTEX.

[20] Lee SY, Sung JH (2018). Gut–liver on a chip toward an in vitro model of hepatic steatosis. Biotechnol. Bioeng..

[21] Müller FA, Sturla SJ (2019). Human in vitro models of nonalcoholic fatty liver disease. Curr. Opin. Toxicol.

[22] Chen WLK (2017). Integrated gut/liver microphysiological systems elucidates inflammatory inter-tissue crosstalk. Biotechnol. Bioeng..

[23] Tsamandouras N (2017). Integrated gut and liver microphysiological systems for quantitative in vitro pharmacokinetic studies. AAPS J..

[24] Costa J, Ahluwalia A (2019). Advances and Current Challenges in Intestinal in vitro Model Engineering: A Digest. Front. Bioeng. Biotechnol.

[25] De Gregorio V (2020). Intestine-liver axis on-chip reveals the intestinal protective role on hepatic damage by emulating ethanol first-pass metabolism. Front. Bioeng. Biotechnol..

[26] Jeon JW, Lee SH, Kim D, Sung JH (2021). In vitro hepatic steatosis model based on gut–liver-on-a-chip. Biotechnol. Prog..

[27] Yang J (2022). Gut-liver-axis microphysiological system for studying cellular fluidic shear stress and inter-tissue interaction. Biomicrofluidics.

[28] Wen X, Yoshimoto K, Yamanaka M, Terada S, Kamei K (2021). In vitro nonalcoholic fatty liver disease model with cyclo-olefin-polymer-based microphysiological systems. Organs-on-a-Chip.

[29] Kamei KI (2017). Integrated heart/cancer on a chip to reproduce the side effects of anti-cancer drugs: In vitro. RSC Adv..

[30] Yu ZTF (2009). Integrated microfluidic devices for combinatorial cell-based assays. Biomed. Microdevices.

[31] Kamei KI (2009). An integrated microfluidic culture device for quantitative analysis of human embryonic stem cells. Lab Chip.

[32] Huang B, Wu H, Kim S, Zare RN (2005). Coating of poly(dimethylsiloxane) with n-dodecyl-β-D-maltoside to minimize nonspecific protein adsorption. Lab Chip.

[33] Kamei KI (2013). Phenotypic and transcriptional modulation of human pluripotent stem cells induced by nano/microfabrication materials. Adv. Healthc. Mater..

[34] Regehr KJ (2009). Biological implications of polydimethylsiloxane-based microfluidic cell culture. Lab Chip.

[35] Wong I, Ho CM (2009). Surface molecular property modifications for poly(dimethylsiloxane) (PDMS) based microfluidic devices. Microfluid. Nanofluidics.

[36] Kim HJ, Ingber DE (2013). Gut-on-a-Chip microenvironment induces human intestinal cells to undergo villus differentiation. Integr. Biol..

[37] Kulthong K (2018). Implementation of a dynamic intestinal gut-on-a-chip barrier model for transport studies of lipophilic dioxin congeners. RSC Adv..

[38] Gómez-Lechón MJ (2007). A human hepatocellular in vitro model to investigate steatosis. Chem. Biol. Interact..

[39] Marra F, Svegliati-Baroni G (2018). Lipotoxicity and the gut-liver axis in NASH pathogenesis. J. Hepatol..

[40] Hirsova P, Ibrabim SH, Gores GJ, Malhi H (2016). Thematic review series: Lipotoxicity: Many roads to cell dysfunction and cell death lipotoxic lethal and sublethal stress signaling in hepatocytes: Relevance to NASH pathogenesis. J. Lipid Res..

[41] Ogawa Y (2018). Palmitate-induced lipotoxicity is crucial for the pathogenesis of nonalcoholic fatty liver disease in cooperation with gut-derived endotoxin. Sci. Rep..

[42] McQuin C (2018). CellProfiler 3.0: Next-generation image processing for biology. PLoS Biol..

[43] Carpenter AE (2006). CellProfiler: image analysis software for identifying and quantifying cell phenotypes. Genome Biol..

[44] Sirenko O, Hesley J, Rusyn I, Cromwell EF (2014). High-content high-throughput assays for characterizing the viability and morphology of human iPSC-derived neuronal cultures. Assay Drug Dev. Technol..

[45] Gilbert DF (2011). A novel multiplex cell viability assay for high-throughput RNAi screening. PLoS ONE.

[46] Van Der Maaten L, Hinton G (2008). Visualizing data using t-SNE. J. Mach. Learn. Res..

[47] Caldez MJ, Bjorklund M, Kaldis P (2020). Cell cycle regulation in NAFLD: when imbalanced metabolism limits cell division. Hepatol. Int..

[48] Kulthong K (2020). Microfluidic chip for culturing intestinal epithelial cell layers: characterization and comparison of drug transport between dynamic and static models. Toxicol. Vitr.

[49] Villenave R (2017). Human gut-on-a-chip supports polarized infection of coxsackie B1 virus in vitro. PLoS ONE.

[50] Kim HJ, Huh D, Hamilton G, Ingber DE (2012). Human gut-on-a-chip inhabited by microbial flora that experiences intestinal peristalsis-like motions and flow. Lab Chip.

[51] Delon LC (2019). A systematic investigation of the effect of the fluid shear stress on Caco-2 cells towards the optimization of epithelial organ-on-chip models. Biomaterials.

[52] Du Y (2017). Mimicking liver sinusoidal structures and functions using a 3D-configured microfluidic chip. Lab Chip.

[53] Wang X (2018). Fluid shear stress promotes autophagy in hepatocellular carcinoma cells. Int. J. Biol. Sci..

[54] Camakaris J, Voskoboinik I, Mercer JF (1999). Molecular mechanisms of copper homeostasis. Biochem. Biophys. Res. Commun..

[55] Khan S, Zafar A, Naseem I (2020). Redox cycling of copper by coumarin-di(2-picolyl)amine hybrid molecule leads to ROS-mediated modulation of redox scavengers, DNA damage and cell death in diethylnitrosamine induced hepatocellular carcinoma. Bioorg. Chem..

[56] Parsekar SU (2018). DNA binding, cleavage and cytotoxicity studies of three mononuclear Cu(II) chloro-complexes containing N–S donor Schiff base ligands. J. Biol. Inorg. Chem..

[57] Foo JB (2018). Copper complex derived from S-benzyldithiocarbazate and 3-acetylcoumarin induced apoptosis in breast cancer cell. BioMetals.

[58] Gori M (2020). Palmitic acid affects intestinal epithelial barrier integrity and permeability in vitro. Antioxidants.

[59] Storniolo CE, Cabral M, Busquets MA, Martín-Venegas R, Moreno JJ (2020). Dual behavior of long-chain fatty acids and their cyclooxygenase/lipoxygenase metabolites on human intestinal Caco-2 cell growth. Front. Pharmacol.

[60] Embade N, Valeron PF, Aznar S, Lopez-Collazo E, Lacal JC (2000). Apoptosis induced by Rac GTPase correlates with induction of FasL and ceramides production. Mol. Biol. Cell.

[61] Simon S, Schubert R (2012). Inhibitory effect of phospholipids on P-glycoprotein: cellular studies in Caco-2, MDCKII mdr1 and MDCKII wildtype cells and P-gp ATPase activity measurements. Biochim. Biophys. Acta—Mol. Cell Biol. Lipids.

[62] Berryman S, Matthews K, Lee JH, Duffy SP, Ma H (2020). Image-based phenotyping of disaggregated cells using deep learning. Commun. Biol.

[63] Ramos MJ, Bandiera L, Menolascina F, Fallowfield JA (2022). In vitro models for non-alcoholic fatty liver disease: Emerging platforms and their applications. iScience.

[64] Grouls M (2022). Differential gene expression in iPSC-derived human intestinal epithelial cell layers following exposure to two concentrations of butyrate, propionate and acetate. Sci. Rep..

[65] Green CJ (2020). Studying non-alcoholic fatty liver disease: the ins and outs of in vivo, ex vivo and in vitro human models. Horm. Mol. Biol. Clin. Investig..

[66] Kostrzewski T (2021). Modelling human liver fibrosis in the context of non-alcoholic steatohepatitis using a microphysiological system. Commun. Biol..

[67] Ganji SH, Kashyap ML, Kamanna VS (2015). Niacin inhibits fat accumulation, oxidative stress, and inflammatory cytokine IL-8 in cultured hepatocytes: Impact on non-alcoholic fatty liver disease. Metabolism.

[68] Thomson JA (1998). Embryonic stem cell lines derived from human blastocysts. Science.

[69] Takahashi K (2007). Induction of pluripotent stem cells from adult human fibroblasts by defined factors. Cell.

[70] Yu J (2007). Induced pluripotent stem cell lines derived from human somatic cells. Science.

[71] Lowry WE (2008). Generation of human induced pluripotent stem cells from dermal fibroblasts. Proc. Natl. Acad. Sci. USA.

[72] Makarova KS (2020). Evolutionary classification of CRISPR–Cas systems: a burst of class 2 and derived variants. Nat. Rev. Microbiol.

[73] Yang, J. et al. Multilayered microfluidic device for controllable flow perfusion of gut-liver on a chip. *21st International Conference on Solid-State Sensors*, *Actuators and Microsystems (Transducers*). 176–179 (IEEE, 2021).

[74] van Meer BJ (2017). Small molecule absorption by PDMS in the context of drug response bioassays. Biochem. Biophys. Res. Commun..

[75] Berthier E, Young EWK, Beebe D (2012). Engineers are from PDMS-land, Biologists are from Polystyrenia. Lab Chip.

[76] Huh D (2010). Reconstituting organ-level lung functions on a chip. Science.

[77] Yoshimoto K (2020). Recapitulation of human embryonic heartbeat to promote differentiation of hepatic endoderm to hepatoblasts. Front. Bioeng. Biotechnol..

[78] Wang M (2022). Application of perfluoropolyether elastomers in microfluidic drug metabolism assays. Int. J. Pharm.

[79] Liao S, He Y, Chu Y, Liao H, Wang Y (2019). Solvent-resistant and fully recyclable perfluoropolyether-based elastomer for microfluidic chip fabrication. J. Mater. Chem. A.

[80] Campbell SB (2020). Beyond polydimethylsiloxane: alternative materials for fabrication of organ-on-a-chip devices and microphysiological systems. ACS Biomater. Sci. Eng..

[81] Albillos A, de Gottardi A, Rescigno M (2020). The gut-liver axis in liver disease: pathophysiological basis for therapy. J. Hepatol..

[82] Ma X (2015). Experimental study of numerical optimization for 3-D microstructuring using DMD-based grayscale lithography. J. Microelectromech. Syst..

[83] Kato, Y., Hirai, Y., Kamei, K., Tsuchiya, T. & Tabata, O. Microfluidic device to interconnect multiple organs via fluidic circulation: towards body-on-a-chip. *18th International Conference on Solid-State Sensors, Actuators and Microsystems (Transducers)*. 1549–1552 (IEEE, 2015).

[84] Langmead B, Salzberg SL (2012). Fast gapped-read alignment with Bowtie 2. Nat. Methods.

[85] Dobin A (2013). STAR: Ultrafast universal RNA-seq aligner. Bioinformatics.

[86] Hunt SE (2018). Ensembl variation resources. Database.

[87] Li B, Dewey CN (2011). RSEM: accurate transcript quantification from RNA-Seq data with or without a reference genome. BMC Bioinformatics.

[88] Love MI, Huber W, Anders S (2014). Moderated estimation of fold change and dispersion for RNA-seq data with DESeq2. Genome Biol.

[89] Wang J, Vasaikar S, Shi Z, Greer M, Zhang B (2017). WebGestalt 2017: a more comprehensive, powerful, flexible and interactive gene set enrichment analysis toolkit. Nucleic Acids Res..

[90] Subramanian A (2005). Gene set enrichment analysis: a knowledge-based approach for interpreting genome-wide expression profiles. Proc. Natl. Acad. Sci. USA.

[91] Demšar J (2013). Orange: Data mining toolbox in python. J. Mach. Learn. Res..

