# Supplementary material for: Integrated-gut-liver-on-a-chip platform as an in vitro human model of non-alcoholic fatty liver disease
Source: Commun Biol. 2023 Mar 23;6:310. doi: 10.1038/s42003-023-04710-8 (PMC10036655; doi:10.1038/s42003-023-04710-8)
Supplement: Supplementary file 1 — Supplementary Information [file 42003_2023_4710_MOESM1_ESM.pdf]

Supplementary Information for

Integrated gut–liver-on-a-chip platform as an *in vitro* human model of non-alcoholic fatty liver disease

Jiandong Yang[1], Yoshikazu Hirai[1,2*], Kei Iida[3,4], Shinji Ito[4], Marika Trumm[1,5,6], Shiho Terada[5], Risako Sakai[5], Toshiyuki Tsuchiya[1], Osamu Tabata[1,5,7], and Ken-ichiro Kamei[5,8,9,10*]

[1]*Department of Micro-Engineering, Kyoto University, Kyotodaigaku-Katsura, Nishikyo-ku, Kyoto 615-8540, JAPAN*

[2]*Department of Mechanical Engineering and Science, Kyoto University, Kyotodaigaku-Katsura, Nishikyo-ku, Kyoto 615-8540, JAPAN*

[3]*Medical Research Support Center, Graduate School of Medicine, Kyoto University, Yoshida Konoe-cho, Kyoto 606-8501, JAPAN*

[4]*Faculty of Science and Engineering, Kindai University, 3-4-1 Kowakae, Higashiosaka, Osaka 577-8502, JAPAN*

[5]*Institute for Integrated Cell-Material Sciences, Kyoto University, Yoshida-Ushinomiya-cho, Sakyo-ku, Kyoto 606-8501, JAPAN*

[6]*Institute for Pharmacy and Molecular Biotechnology, Heidelberg University, Heidelberg, 69120, GERMANY*

[7]*Faculty of Engineering/Graduate School of Engineering, Kyoto University of Advanced Science, Gotanda-cho, Yamanouchi, Ukyo-ku, Kyoto, 615-8577, JAPAN*

[8]*Wuya College of Innovation, Shenyang Pharmaceutical University, Liaoning 110016, China*

[9]*Department of Pharmaceutics, Shenyang Pharmaceutical University, Liaoning 110016, China*

[10]*Programs of Biology and Bioengineering, Divisions of Science and Engineering, New York University Abu Dhabi, Abu Dhabi, UAE*

*Corresponding authors.

E-mail addresses: hirai@me.kyoto-u.ac.jp (YH), kamei.kenichiro.7r@kyoto-u.ac.jp (KK)

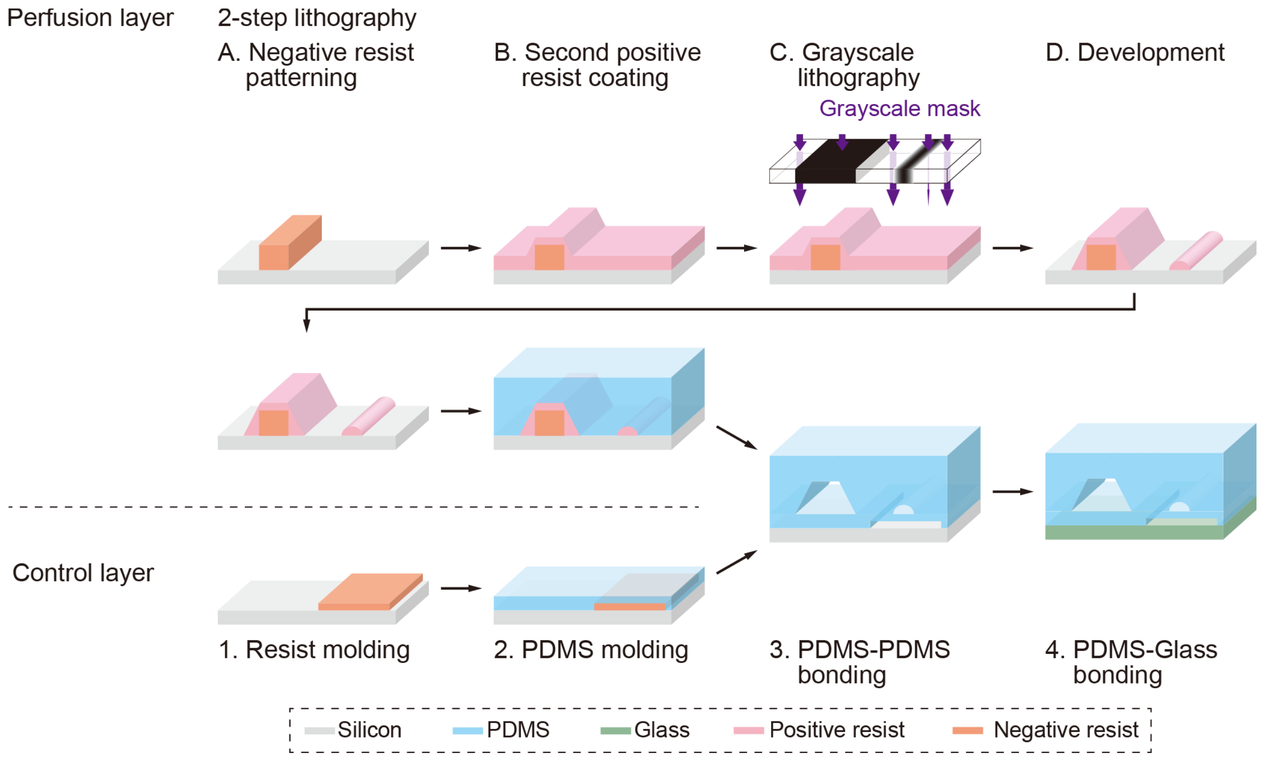

Supplementary Fig. S1 Fabrication procedure for the iGLC platform.

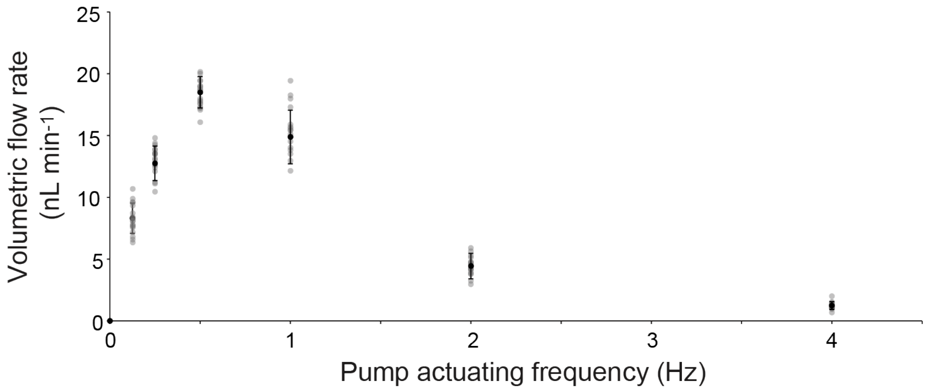

Supplementary Fig. S2 Flow rates of the cell culture medium in the iGLC platform operated by the integrated micro-pump. The flow rates were regulated by the actuation frequency for the sequential opening and closing of a set of three micro-valves acting as a micro-pump. To evaluate the flow rates, micro-beads (4.5 μm in diameter; Polysciences, Inc.) were used to visualize the medium flow and measure the flow distance. Prior to measurements, the microfluidic channel was coated with 1% (w/v) bovine serum albumin (BSA, Sigma-Aldrich) in PBS for 2 h at 25 °C to prevent non-specific adhesion on the channel. Micro-beads were suspended in 1% (w/v) BSA solution at 1.0×10^6 beads mL^{-1}. Then, 5 μL of bead-containing solution was introduced into the chip. After a stable flow was reached with the pump, the moving distance of the micro-beads were measured. The average flow velocity was half the micro-bead velocity at the center line. The volumetric flow rates were calculated by multiplying the flow velocity with the cross-sectional area of the microfluidic channel. Each dot represents the mean ± standard deviation (n = 18).

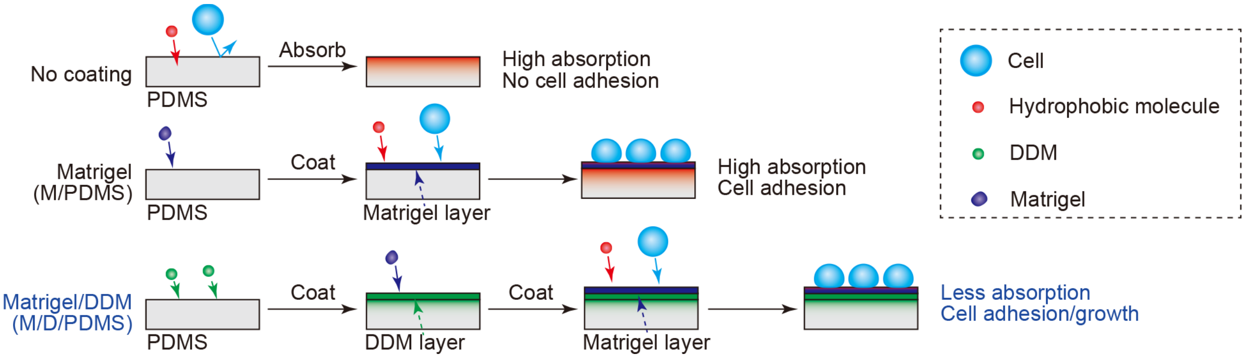

Supplementary Fig. S3 Schematic for PDMS coating with DDM and Matrigel to respectively prevent absorption of hydrophobic molecules in PDMS and promote cell adhesion and growth on PDMS.

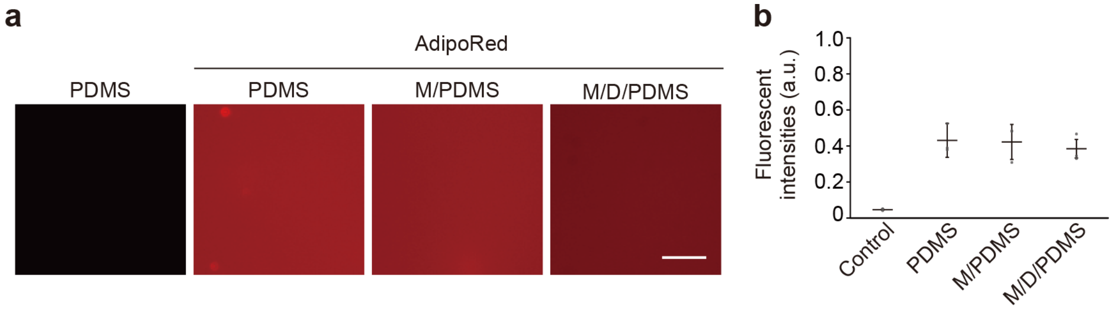

Supplementary Fig. S4 Absorption of hydrophobic molecules in PDMS. a, Fluorescent micrographs of PDMS coated with Matrigel (M/PDMS), Matrigel and DDM (M/D/PDMS), and PDMS without any coating (PDMS) that were treated with the AdipoRed lipid fluorescent dye. PDMS without any coating or treatment was used as the control. The scale bar represents 100 µm. **b,** Fluorescent intensities of AdipoRed dye absorbed in PDMS based on the microscopic photographs shown in **a**. The bars and error bars represent the mean ± standard deviations (n = 3).

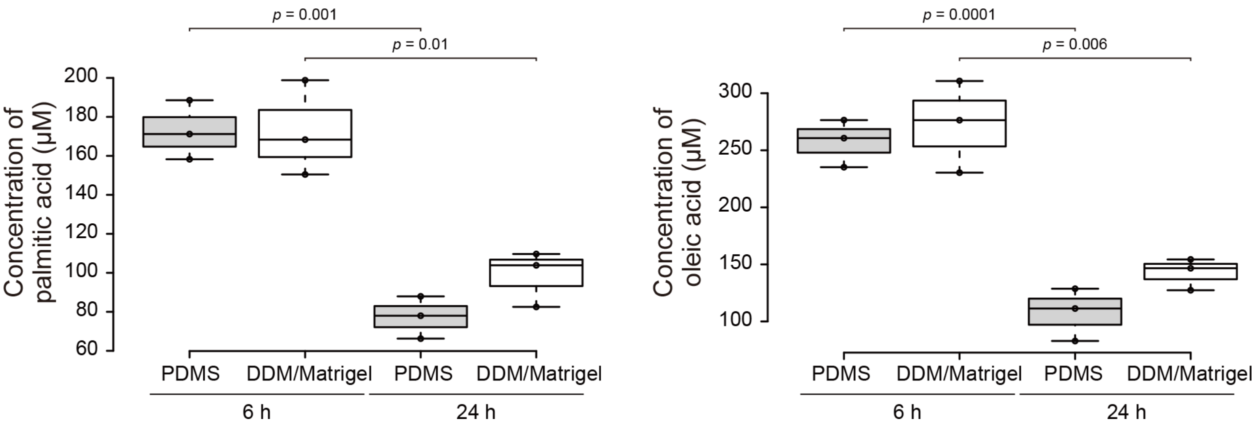

Supplementary Fig. S5 Lipid absorption in PDMS. FFA concentrations [left: palmitic acid (initial: 167 µM); right: oleic acid (initial: 333 µM)] in cell culture media after incubation in PDMS or DDM/Matrigel-coated microfluidic channels for 6 and 24 h. The center lines show the medians. The box limits indicate the 25th and 75th percentiles. The whiskers extend 1.5 times the interquartile range from the maximum and minimum points; data points are plotted as circles. *p* values were estimated with Student's *t*-test (n = 3).

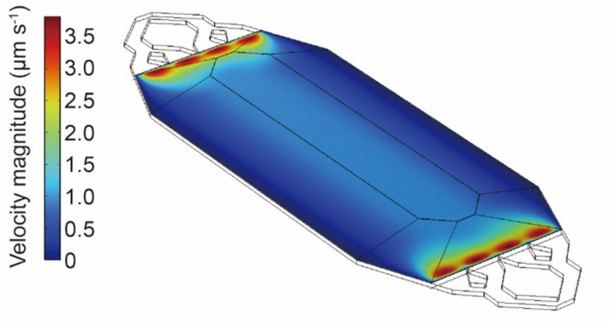

Supplementary Fig. S6 Simulated flow field profile in the cell culture chamber. Cell culture chamber flow profile was shown in a 0 to 1 μm s^{-1} flow velocity ranges; the estimated mean fluid shear stress on cells was around 3.53×10^{-4} dyne cm^{-2}.

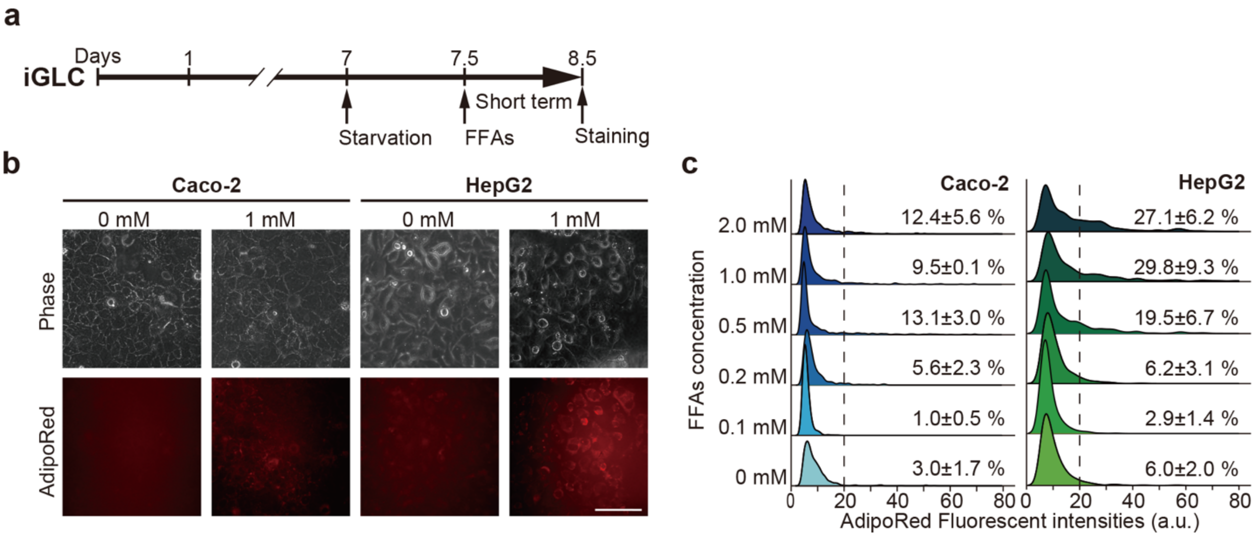

Supplementary Fig. S7 Evaluation of FFAs accumulation in Caco-2 and HepG2 cells. a, Experimental procedure to induce NAFLD in an iGLC platform. **b,** Phase contrast and fluorescent micrographs of Caco-2 and HepG2 cells treated with FFAs (0 and 1 mM) stained with AdipoRed lipid fluorescent dye. Scale bars represent 100 µm. **c,** Ridgeline plots to evaluate FFA accumulation in individual cells [Caco-2 (*left*) and HepG2 (*right*)] after FFA treatment for 24 h. The *p*-values were estimated with the Tukey–Kramer test and are presented in **Supplementary Tables S3** and **S4** for the Caco-2 and HepG2 cells, respectively. Cells with over 20 of AdipoRed fluorescent intensity were considered as lipid accumulated cells. The data represent the mean ± standard deviation (n=3).

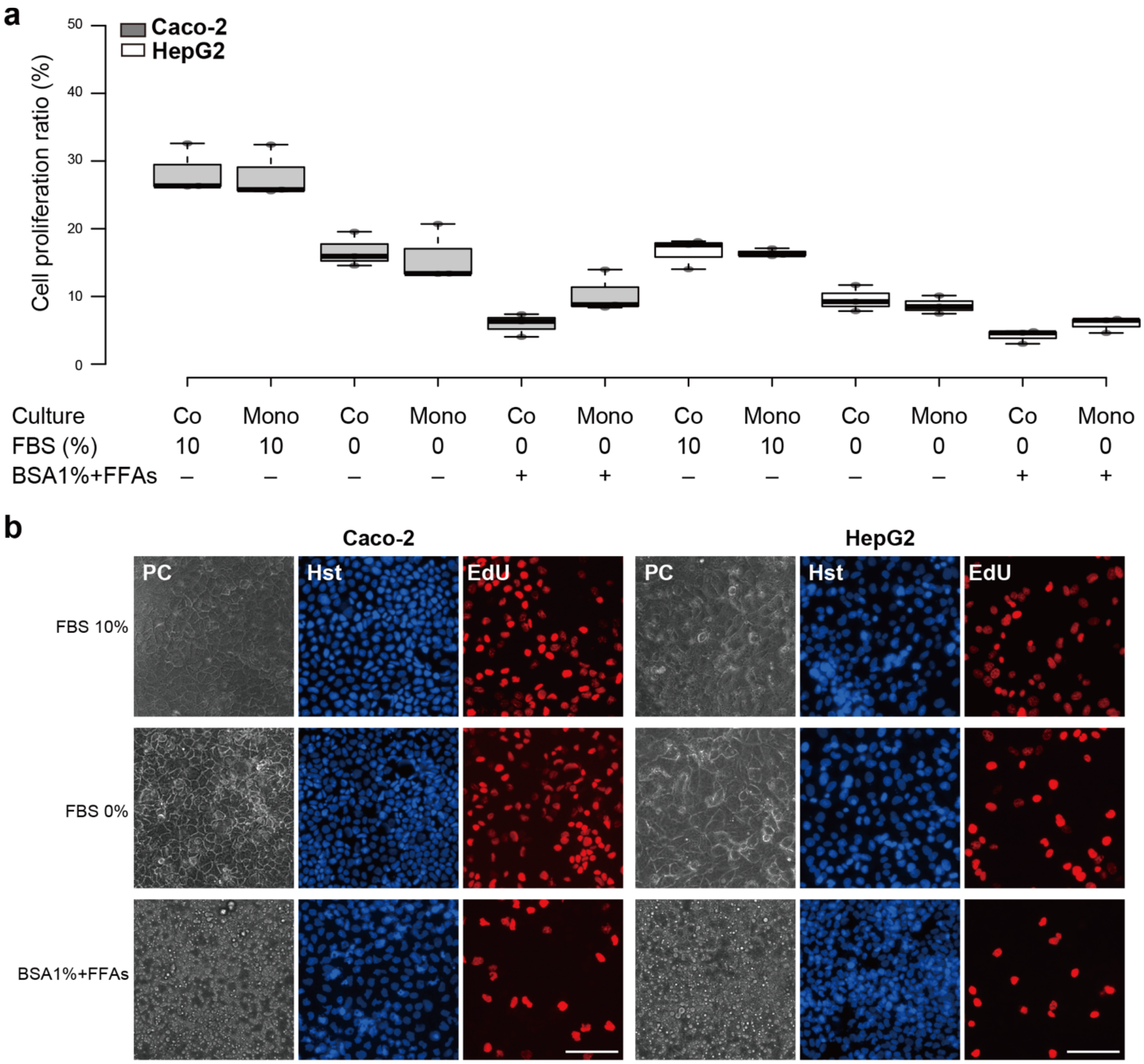

Supplementary Fig. S8 Evaluation of cell proliferation of Caco-2 and HepG2 cells in iGLC. **a,** Box plot to evaluate cell proliferation of Caco-2 and HepG2 cells in DMEM cell culture with 10% FBS or 1% BSA (contained with 1 mM free fatty acids; FFAs), under co- or (Co) or mono-culture (Mono) conditions for 7 days. **b,** longer-term maintaining the cell culture of Caco-2 and HepG2 cells. The Caco-2 and HepG2 cells were co-cultured on the chips, and cell morphology was monitored every day. The cell proliferation was tested by Click-iT™ EdU Cell Proliferation Kit. Hst: Hoechst 33342; EdU: EdU Cell kit, Alexa Fluor™ 647 dye. The scale bar represents 100 µm. The center lines show the medians. The box limits indicate the 25th and 75th percentiles. The whiskers extend 1.5 times the interquartile range from the maximum and minimum points; data points are plotted as circles (n = 3).

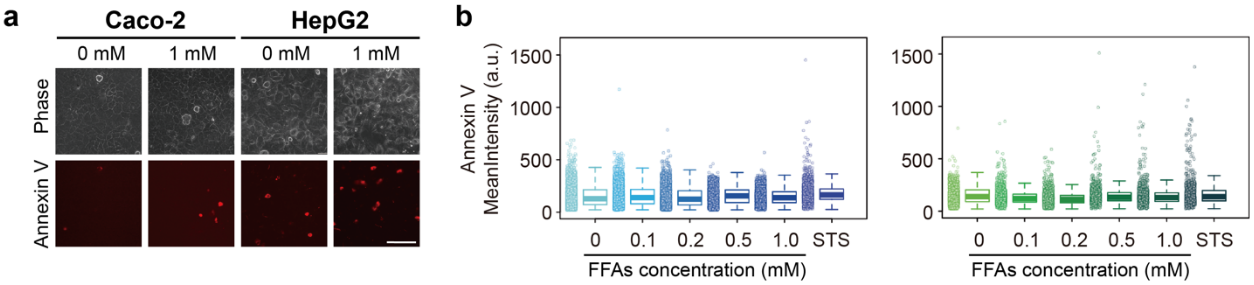

Supplementary Fig. S9 FFAs treatment for 1 day did not affect apoptotic cellular status. a, Phase contrast and fluorescent micrographs of Caco-2 and HepG2 cells treated with FFAs (0 and 1 mM) stained with the Annexin V apoptotic cell marker. The scale bars represent 100 µm. **b,** Box plots to evaluate individual apoptotic cells [Caco-2 (left) and HepG2 (right)] after FFA treatment for 24 h. For comparison, cells were treated with 1 µM of staurosporine (STS) for 24 h. The center lines of the boxplots show the medians. The box limits indicate the 25th and 75th percentiles. The whiskers extend 1.5 times the interquartile range from the 25th and 75th percentiles.

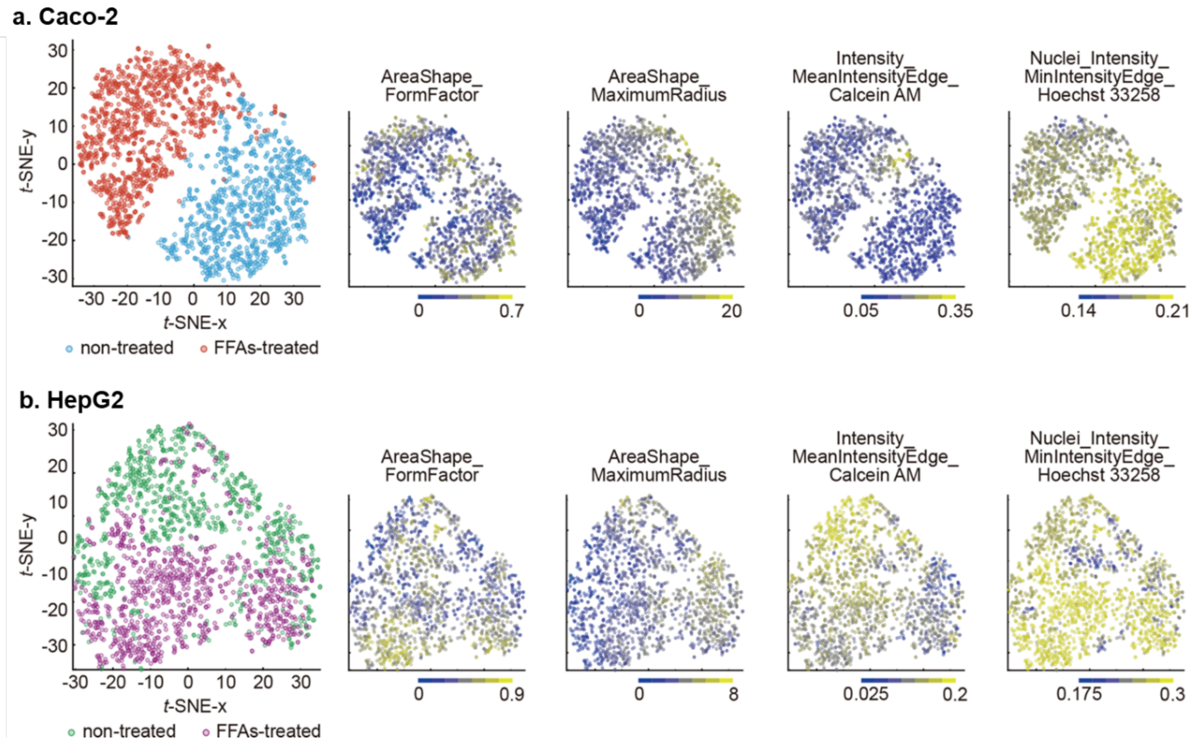

Supplementary Fig. S10 Single-cell profiling cell viability and t-SNE analyses of the 1-day free fatty acids treatment. a, b, Two-dimensional t-SNE plots of microscopic single-cell profiling of Caco-2 (c) and HepG2 (d) treated with 1 mM of FFAs or no treatment and stained with Calcein AM cellular and Hoechst 33258 nuclei markers.

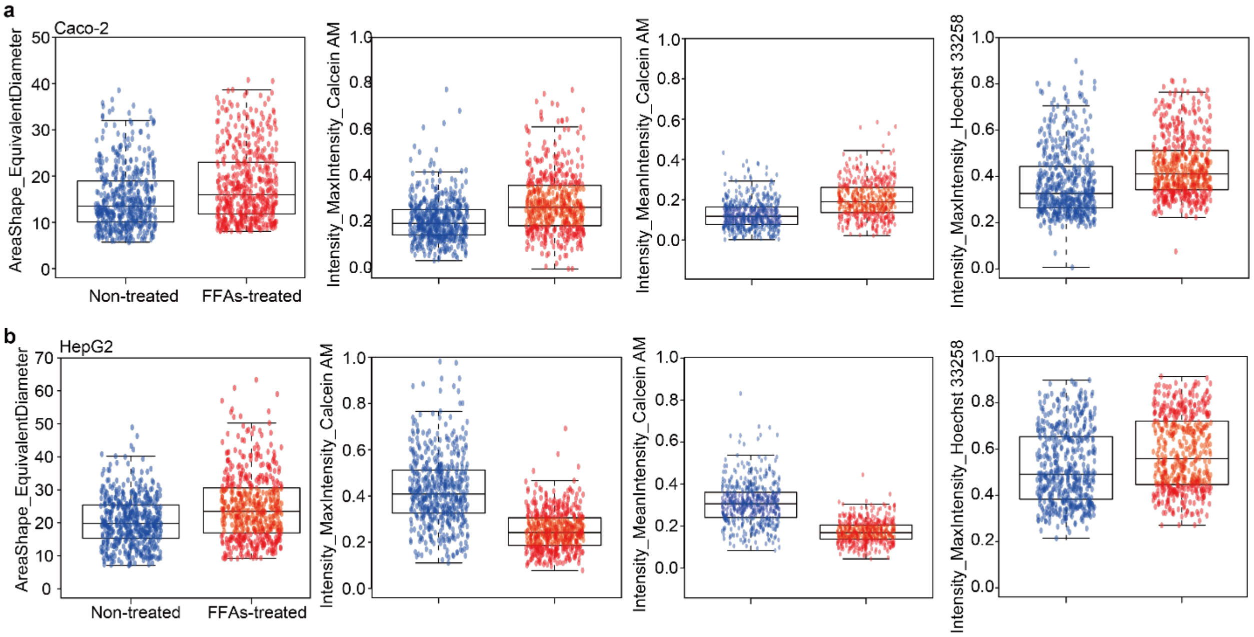

Supplementary Fig. S11 Boxplots comparing cellular parameters of non-treated and FFAs-treated Caco-2 and HepG2 cells of 7 days FFAs treatment. The centrelines of the boxplots show the medians. The box limits indicate the 25th and 75th percentiles. The whiskers extend 1.5 times the interquartile range from the 25th and 75th percentiles. *p*-values were estimated with the Tukey-Kramer test and are presented in **Supplementary Table S11 and Table S12**.

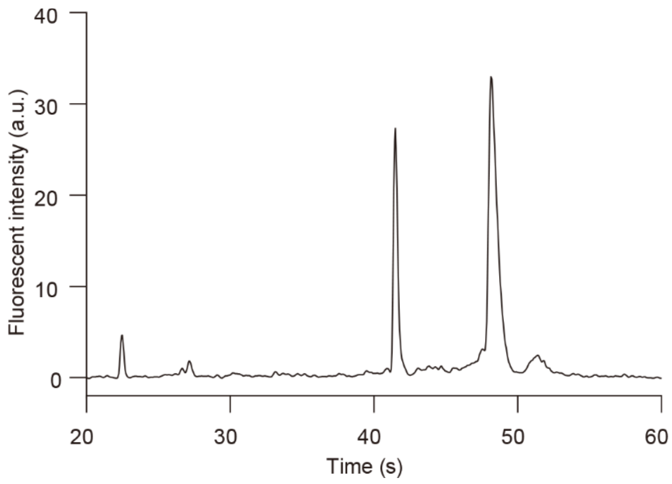

Supplementary Fig. S12 Typical electropherogram of total RNA obtained from Caco-2 cells cultured on a chip with Agilent 2100 Bioanalyzer. The electropherogram shows that 18S and 28S ribosomal RNA (rRNA) bands were clearly detected without smeared bands, and the RNA integrity number (RIN), which is used to standardize RNA quality control, was over 7.0. This result indicates that the total RNA harvested from the chip was sufficient to be applied to RNA sequencing.

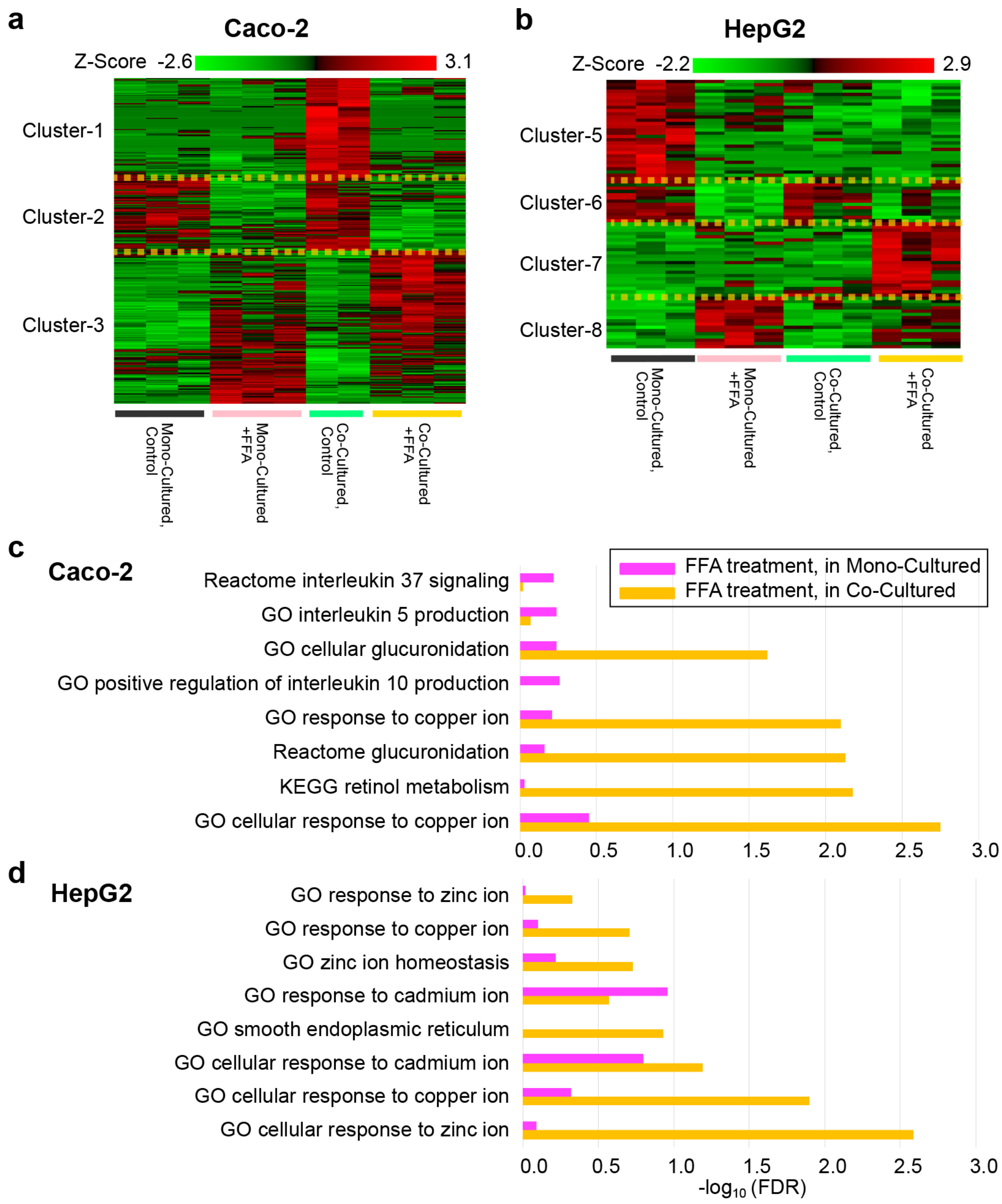

Supplementary Fig. S13 Gene expressions of the effects of FFA treatment for 1 day and crosstalk with the *in vitro* human GLA model. a, b, Heat maps for the DEGs obtained from the 1-day FFAs-treated Caco-2 (**a**) and HepG2 (**b**) experimental sets. Z-values of the expression profiles are shown. **c, d,** Bar charts showing gene enrichment related to certain GO terms and pathways for FFA-treated Caco-2 (**c**) and HepG2 (**d**) cells under mono- and co-cultured conditions.

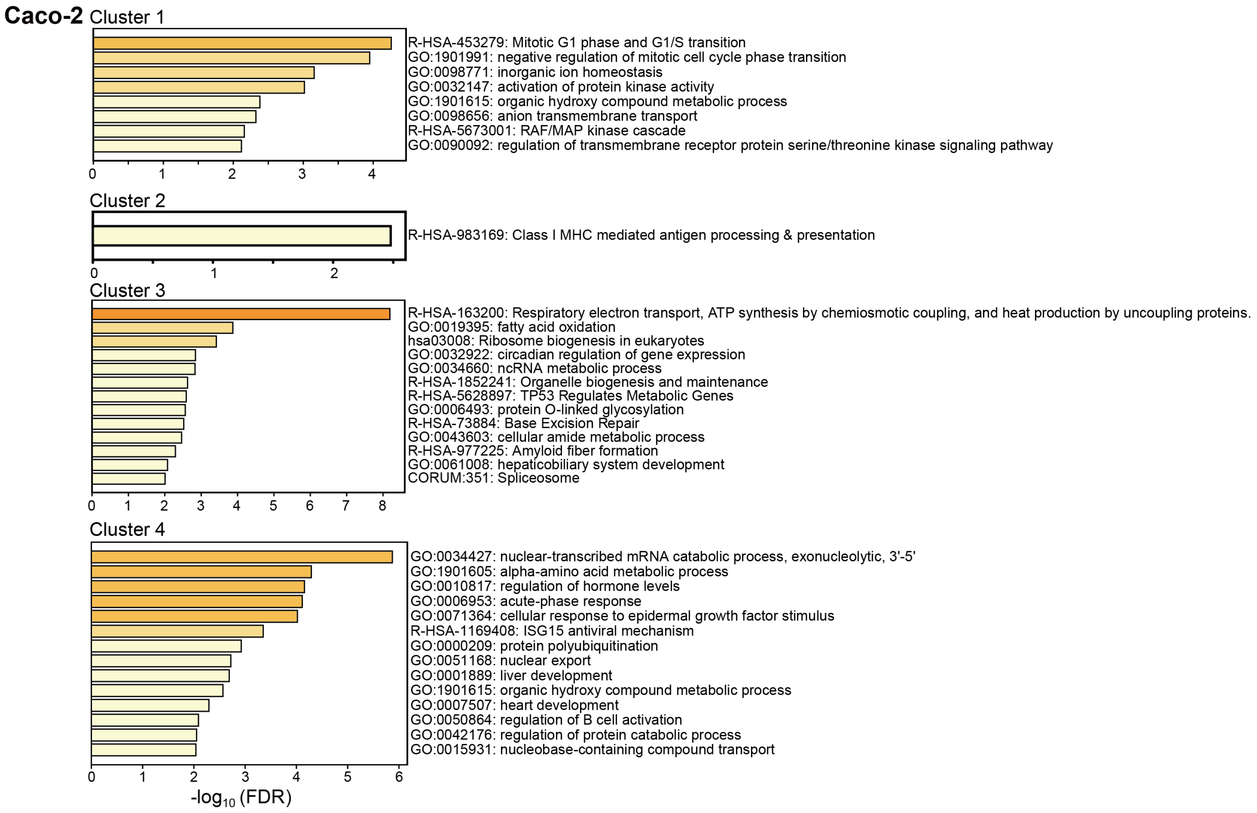

Supplementary Fig. S14 Gene-ontology (GO) terms of Clusters 1 to 4 for Caco-2 cells

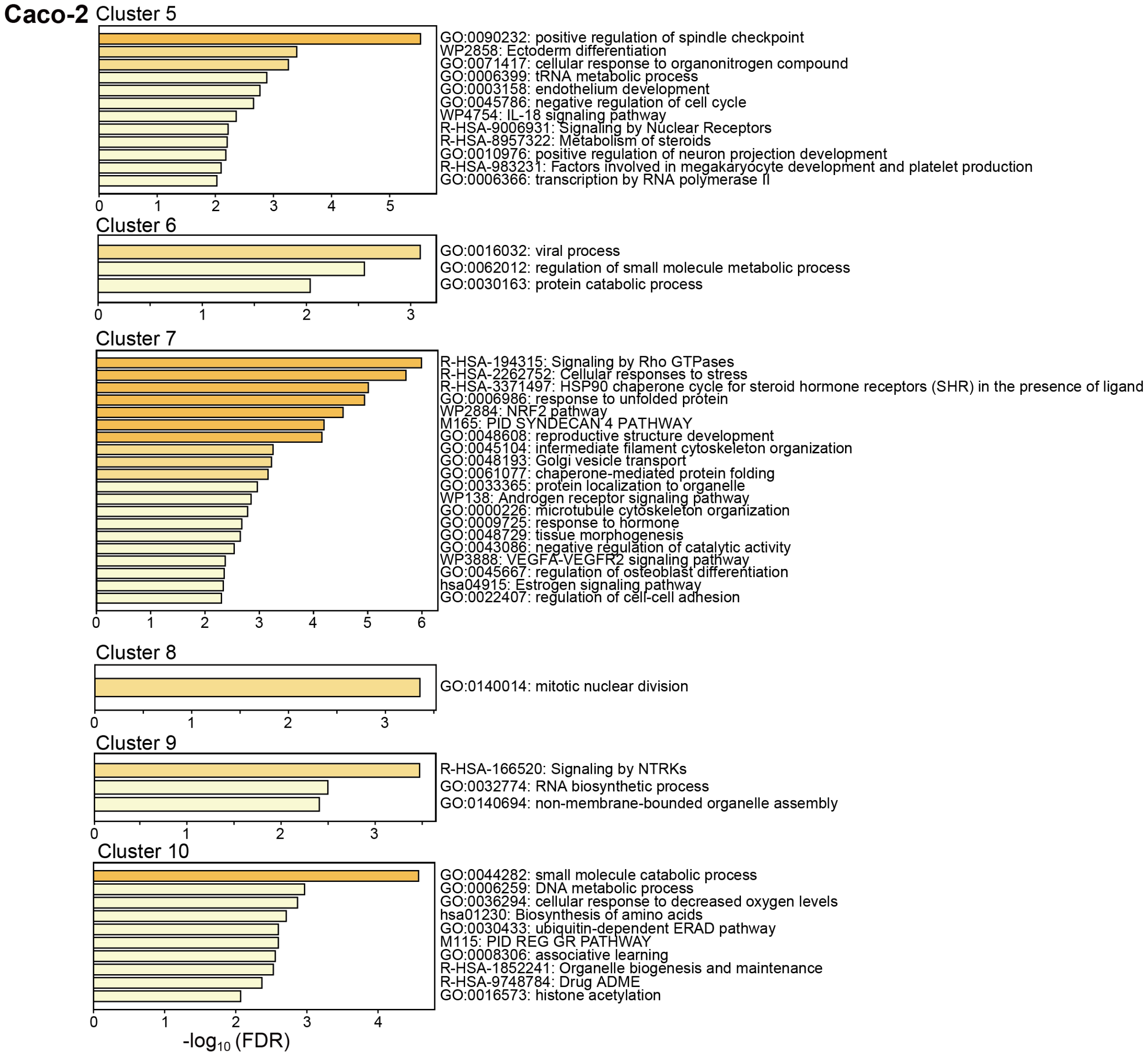

Supplementary Fig. S15 Gene-ontology (GO) terms of Clusters 5 to 10 for Caco-2 cells

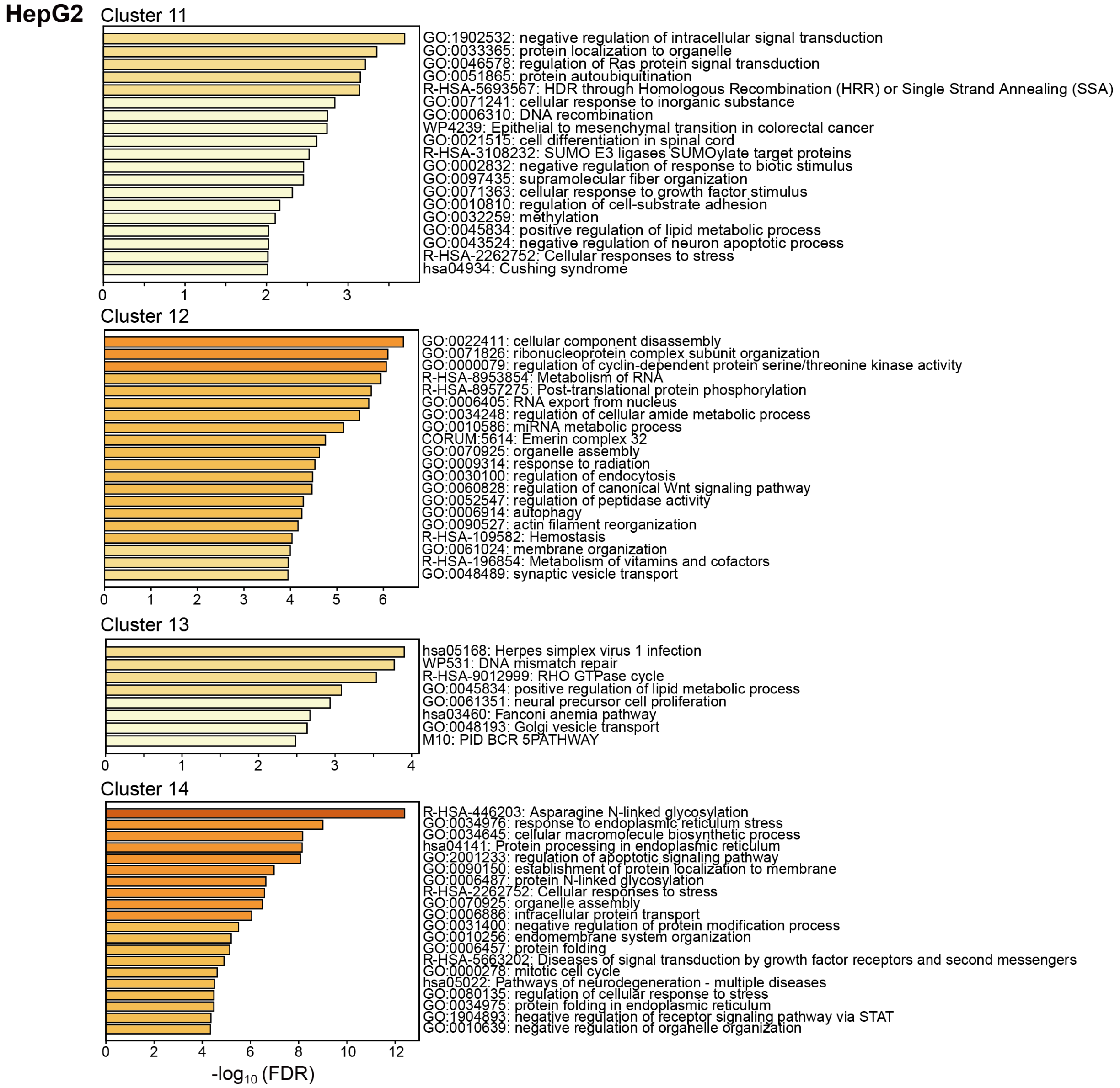

Supplementary Fig. S16 Gene-ontology (GO) terms of Clusters 11 to 14 for HepG2 cells

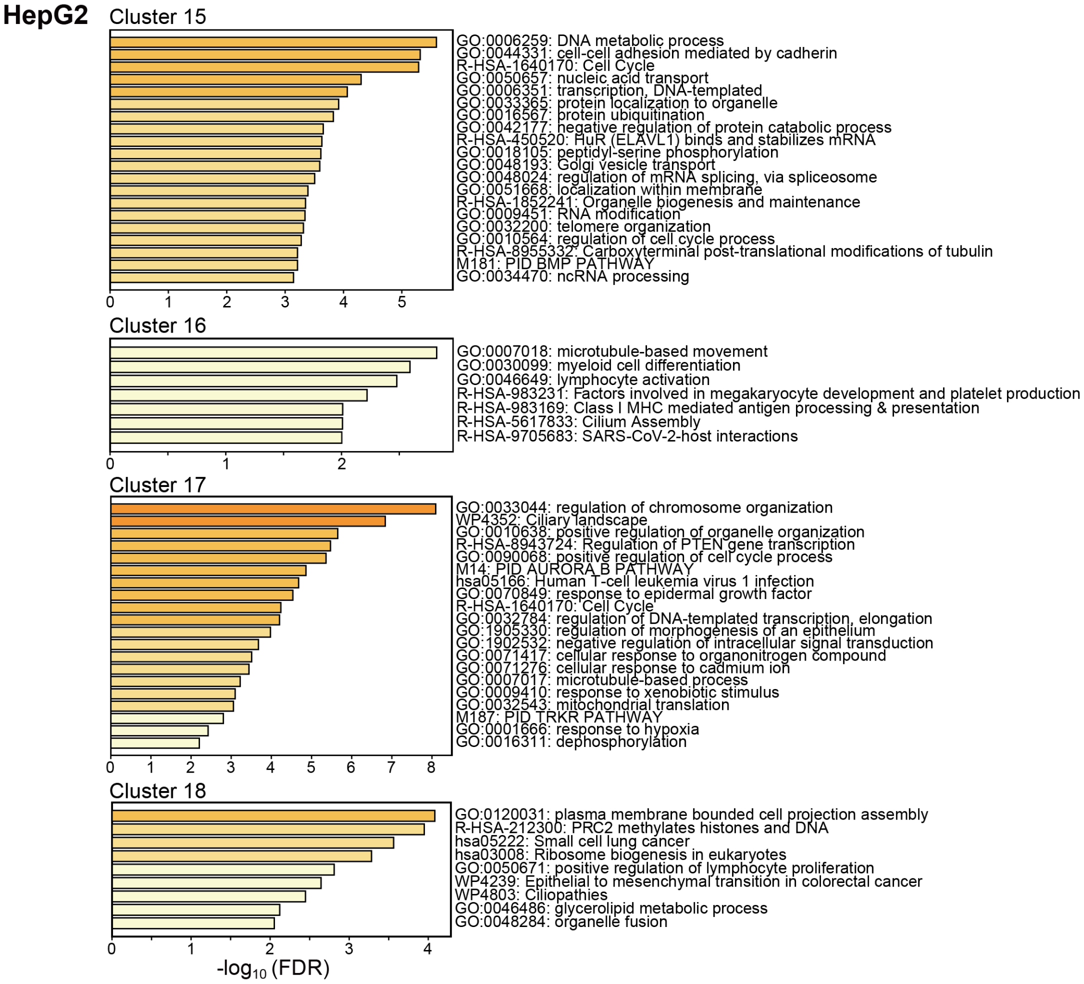

Supplementary Fig. S17 Gene-ontology (GO) terms of Clusters 15 to 18 for HepG2 cells

Supplementary Table S1 *p*-values by Tukey-Kramer test for single-cell profiling for viability staining in Caco-2 cells visualized by Calcein AM.

Sample A	Sample B	Lower	Upper	*p* value
C:H CF	C: C CF	-6.112719475	0.385090584	0.097
C:H CF	C: C S	-45.33705147	-40.09039401	0
C: C CF	C: C S	-42.47323702	-37.22657956	0

C: Caco-2; H: HepG2

S: Static condition; CF: Circulated flow

Supplementary Table S2 *p*-values by Tukey-Kramer test for single-cell profiling for viability staining in HepG2 cells visualized by Calcein AM.

Sample A	Sample B	Lower	Upper	*p* value
C:H CF	C: C CF	-9.751347416	5.209697104	0.757
C:H CF	C: C S	-7.954678346	7.010102229	0.988
C: C CF	C: C S	-5.680112927	9.277187123	0.839

C: Caco-2; H: HepG2

S: Static condition; CF: Circulated flow

Supplementary Table S3 *p*-values by Tukey-Kramer test for single-cell profiling of FFAs accumulation in Caco-2 cells for 1-day treatment visualized by AdipoRed staining

Sample A	Sample B	Lower	Upper	*p* value
0.1 mM	0.2 mM	0.223113	4.250708	0.0193
0.1 mM	0.5 mM	3.169775	7.19737	8.99E-09
0.1 mM	0 mM	0.185112	4.212706	0.0229
0.1 mM	1.0 mM	1.250067	5.277662	5.79E-05
0.1 mM	2.0 mM	2.544007	6.571602	1.08E-08
0.2 mM	0.5 mM	0.932865	4.960459	0.000443
0.2 mM	0 mM	-2.0518	1.975796	1
0.2 mM	1.0 mM	-0.98684	3.040751	0.694
0.2 mM	2.0 mM	0.307096	4.334691	0.0131
0.5 mM	0 mM	-4.99846	-0.97087	0.000351
0.5 mM	1.0 mM	-3.93351	0.094089	0.0719
0.5 mM	2.0 mM	-2.63957	1.388029	0.95
0 mM	1.0 mM	-0.94884	3.078753	0.659
0 mM	2.0 mM	0.345098	4.372693	0.0109
1.0 mM	2.0 mM	-0.71986	3.307737	0.445

Supplementary Table S4 *p*-values by Tukey-Kramer test for single-cell profiling of FFAs accumulation in HepG2 cells for 1-day treatment visualized by AdipoRed staining

Sample A	Sample B	Lower	Upper	*p* value
0.1 mM	0.2 mM	-0.15943	4.633305	0.0834
0.1 mM	0.5 mM	3.120136	7.912872	0
0.1 mM	0 mM	-1.11966	3.673077	0.652
0.1 mM	1 mM	8.310987	13.10372	0
0.1 mM	2 mM	5.696866	10.4896	0
0.2 mM	0.5 mM	0.883199	5.675935	0.00136
0.2 mM	0 mM	-3.3566	1.436141	0.863
0.2 mM	1 mM	6.07405	10.86679	0
0.2 mM	2 mM	3.459929	8.252665	0
0.5 mM	0 mM	-6.63616	-1.84343	7.14E-06
0.5 mM	1 mM	2.794483	7.587219	9.94E-09
0.5 mM	2 mM	0.180361	4.973097	0.0266
0 mM	1 mM	7.034278	11.82701	0
0 mM	2 mM	4.420156	9.212892	0
1 mM	2 mM	-5.01049	-0.21775	0.0232

Supplementary Table S5 *p*-values by Tukey-Kramer test for single-cell profiling of 7 days FFAs accumulation in Caco-2 cells visualized by AdipoRed staining

Sample A	Sample B	Lower	Upper	*p* value
0 mM-co	0 mM-mono	-1.29137	0.194586	0.23
0 mM-co	1 mM-co	5.379399	6.865359	2.41E-11
0 mM-co	1 mM-mono	6.835213	8.321173	2.41E-11
0 mM-mono	1 mM-co	5.927792	7.413752	2.41E-11
0 mM-mono	1 mM-mono	7.383607	8.869567	2.41E-11
1 mM-co	1 mM-mono	0.712835	2.198795	2.92E-06

Supplementary Table S6 *p*-values by Tukey-Kramer test for single-cell profiling of 7 days FFAs accumulation in HepG2 cells visualized by AdipoRed staining

Sample A	Sample B	Lower	Upper	*p* value
0 mM-co	0 mM-mono	-1.20778	0.646492	0.865
0 mM-co	1 mM-co	4.899882	6.754151	2.41E-11
0 mM-co	1 mM-mono	2.047212	3.90148	2.41E-11
0 mM-mono	1 mM-co	5.180524	7.034793	2.41E-11
0 mM-mono	1 mM-mono	2.327854	4.182122	2.41E-11
1 mM-co	1 mM-mono	-3.7798	-1.92554	2.42E-11

Supplementary Table S7 *p*-values by Tukey-Kramer test for single-cell profiling of apoptotic cells in 7-days FFAs-treated Caco-2 cells visualized by Annexin V staining

Sample A	Sample B	Lower	Upper	*p* value
0 mM-Co	0 mM-Mono	-0.03047	-0.01876	0
0 mM-Co	1 mM-Co	-0.01207	-0.00036	0.031
0 mM-Co	1 mM-Mono	0.054332	0.066037	0
0 mM-Co	STS	0.000587	0.012293	0.0226
0 mM-Mono	1 mM-Co	0.012549	0.024254	0
0 mM-Mono	1 mM-Mono	0.078947	0.090653	0
0 mM-Mono	STS	0.025203	0.036908	0
1 mM-Co	1 mM-Mono	0.060546	0.072251	0
1 mM-Co	STS	0.006801	0.018507	2.13E-08
1 mM-Mono	STS	-0.0596	-0.04789	0

Supplementary Table S8 *p*-values by Tukey-Kramer test for single-cell profiling of apoptotic cells in 7-days FFAs-treated HepG2 cells visualized by Annexin V staining

Sample A	Sample B	Lower	Upper	*p* value
0 mM-Co	0 mM-Mono	-0.004368062	0.012056966	0.705
0 mM-Co	1 mM-Co	-0.002436683	0.013988345	0.307
0 mM-Co	1 mM-Mono	0.095574898	0.111999926	0
0 mM-Co	STS	0.019753004	0.036178032	0
0 mM-Mono	1 mM-Co	-0.006281135	0.010143893	0.968
0 mM-Mono	1 mM-Mono	0.091730446	0.108155474	0
0 mM-Mono	STS	0.015908552	0.03233358	0
1 mM-Co	1 mM-Mono	0.089799067	0.106224095	0
1 mM-Co	STS	0.013977173	0.030402201	0
1 mM-Mono	STS	-0.084034408	-0.06760938	0

Supplementary Table S9 *p*-values by Tukey-Kramer test for single-cell profiling of albumin expression in 7-days FFAs-treated HepG2 cells visualized by albumin staining

Sample A	Sample B	Lower	Upper	*p* value
0 mM-Co	0 mM-Mono	-14.3585	-8.82408	0
0 mM-Co	1 mM-Co	-2.47111	3.06336	0.993
0 mM-Co	1 mM-Mono	-9.963	-4.42854	0
0 mM-Mono	1 mM-Co	9.120202	14.65467	0
0 mM-Mono	1 mM-Mono	1.628305	7.162773	0.000263
1 mM-Co	1 mM-Mono	-10.2591	-4.72466	0

Supplementary Table S10 68 types of Cellular parameters for quantitative cellular phenotype analysis with Calcein AM and Hoechst 33258 staining

AreaShape_Area
AreaShape_BoundingBoxArea
AreaShape_BoundingBoxMaximum_X
AreaShape_BoundingBoxMaximum_Y
AreaShape_BoundingBoxMinimum_X
AreaShape_BoundingBoxMinimum_Y
AreaShape_Center_X
AreaShape_Center_Y
AreaShape_Compactness
AreaShape_Eccentricity
AreaShape_EquivalentDiameter
AreaShape_Extent
AreaShape_FormFactor
AreaShape_MajorAxisLength
AreaShape_MaxFeretDiameter
AreaShape_MaximumRadius
AreaShape_MeanRadius
AreaShape_MedianRadius
AreaShape_MinFeretDiameter
AreaShape_MinorAxisLength
AreaShape_Orientation
AreaShape_Perimeter
AreaShape_Solidity
Intensity_IntegratedIntensityEdge_Hoechst 33258
Intensity_IntegratedIntensityEdge_Calcein AM
Intensity_IntegratedIntensity_Hoechst 33258
Intensity_IntegratedIntensity_Calcein AM
Intensity_LowerQuartileIntensity_Hoechst 33258
Intensity_LowerQuartileIntensity_Calcein AM
Intensity_MADIntensity_Hoechst 33258
Intensity_MADIntensity_Calcein AM
Intensity_MassDisplacement_Hoechst 33258
Intensity_MassDisplacement_Calcein AM
Intensity_MaxIntensityEdge_Hoechst 33258
Intensity_MaxIntensityEdge_Calcein AM
Intensity_MaxIntensity_Hoechst 33258
Intensity_MaxIntensity_Calcein AM

Intensity_MeanIntensityEdge_Hoechst 33258
Intensity_MeanIntensityEdge_Calcein AM
Intensity_MeanIntensity_Hoechst 33258
Intensity_MeanIntensity_Calcein AM
Intensity_MedianIntensity_Hoechst 33258
Intensity_MedianIntensity_Calcein AM
Intensity_MinIntensityEdge_Hoechst 33258
Intensity_MinIntensityEdge_Calcein AM
Intensity_MinIntensity_Hoechst 33258
Intensity_MinIntensity_Calcein AM
Intensity_StdIntensityEdge_Hoechst 33258
Intensity_StdIntensityEdge_Calcein AM
Intensity_StdIntensity_Hoechst 33258
Intensity_StdIntensity_Calcein AM
Intensity_UpperQuartileIntensity_Hoechst 33258
Intensity_UpperQuartileIntensity_Calcein AM
NucleiAreaShape_Area
NucleiAreaShape_Compactness
NucleiAreaShape_Eccentricity
NucleiAreaShape_EulerNumber
NucleiAreaShape_Extent
NucleiAreaShape_FormFactor
NucleiAreaShape_MajorAxisLength
NucleiAreaShape_MaxFeretDiameter
NucleiAreaShape_MaximumRadius
NucleiAreaShape_MeanRadius
NucleiAreaShape_MedianRadius
NucleiAreaShape_MinFeretDiameter
NucleiAreaShape_MinorAxisLength
NucleiAreaShape_Perimeter
NucleiAreaShape_Solidity

Supplementary Table S11 *p*-values by Tukey-Kramer test for single-cell profiling of FFAs-treated Caco-2 cells with the most distinguishable cellular parameters based on Calcein AM and Hoechst 33258 staining

Items	A	B	Lower	Upper	*p* value
AreaShape_EquivalentDiameter	FFAs-	Non-	-2.36536	-0.56856	0.0014
Intensity_MaxIntensity_Calcein AM	FFAs-	Non-	-0.10853	-0.08029	0
Intensity_MeanIntensity_Calcein AM	FFAs-	Non-	-0.11294	-0.09258	0
Intensity_MaxIntensity_Hoechst 33258	FFAs-	Non-	-0.086	-0.05159	0

Supplementary Table S12 *p*-values by Tukey-Kramer test for single-cell profiling of FFAs-treated HepG2 cells with the most distinguishable cellular parameters based on Calcein AM and Hoechst 33258 staining

Items	A	B	Lower	Upper	*p* value
AreaShape_EquivalentDiameter	FFAs-	Non-	-4.44339	-2.25715	2.53E-09
Intensity_MaxIntensity_Calcein AM	FFAs-	Non-	0.15985	0.190296	0
Intensity_MeanIntensity_Calcein AM	FFAs-	Non-	0.1563806	0.1764105	0
Intensity_MaxIntensity_Hoechst 33258	FFAs-	Non-	-0.04341	-0.00386	0.0192
