# Supplementary material for: Integrated-gut-liver-on-a-chip platform as an in vitro human model of non-alcoholic fatty liver disease
Source: Commun Biol. 2023 Mar 23;6:310. doi: 10.1038/s42003-023-04710-8 (PMC10036655; doi:10.1038/s42003-023-04710-8)
Supplement: Supplementary file 2 — Description of Additional Supplementary Files [file 42003_2023_4710_MOESM2_ESM.pdf]

Description of Additional Supplementary Files

File name: Supplementary Data 1

Description: Source data underlying the graph Figure 2d presented in the main manuscript

File name: Supplementary Data 2

Description: Source data underlying the graph Figure 3b presented in the main manuscript

File name: Supplementary Data 3

Description: Source data underlying the graph Figure 4b and 4d presented in the main manuscript

File name: Supplementary Data 4

Description: Source data underlying the graph Figure 5 presented in the main manuscript

File name: Supplementary Data 5

Description: Differentially expressed genes (DEGs) in Caco-2 cells identified by ANOVA.

File name: Supplementary Data 6

Description: Differentially expressed genes (DEGs) in HepG2 cells identified by ANOVA.
