# Supplementary material for: Integrated-gut-liver-on-a-chip platform as an in vitro human model of non-alcoholic fatty liver disease
Source: Commun Biol. 2023 Mar 23;6:310. doi: 10.1038/s42003-023-04710-8 (PMC10036655; doi:10.1038/s42003-023-04710-8)
Supplement: Supplementary file 9 — Reporting Summary [file 42003_2023_4710_MOESM9_ESM.pdf]

nature research

Corresponding author(s): Hirai Yoshikazu & Ken-ichiro Kamei

Last updated by author(s): Feb 19, 2023

Reporting Summary

Nature Research wishes to improve the reproducibility of the work that we publish. This form provides structure for consistency and transparency in reporting. For further information on Nature Research policies, see our Editorial Policies and the Editorial Policy Checklist.

Statistics

For all statistical analyses, confirm that the following items are present in the figure legend, table legend, main text, or Methods section.

n/a	Confirmed	
☐	☒	The exact sample size (*n*) for each experimental group/condition, given as a discrete number and unit of measurement
☐	☒	A statement on whether measurements were taken from distinct samples or whether the same sample was measured repeatedly
☐	☒	The statistical test(s) used AND whether they are one- or two-sided<br>*Only common tests should be described solely by name; describe more complex techniques in the Methods section.*
☐	☒	A description of all covariates tested
☐	☒	A description of any assumptions or corrections, such as tests of normality and adjustment for multiple comparisons
☐	☒	A full description of the statistical parameters including central tendency (e.g. means) or other basic estimates (e.g. regression coefficient) AND variation (e.g. standard deviation) or associated estimates of uncertainty (e.g. confidence intervals)
☐	☒	For null hypothesis testing, the test statistic (e.g. *F*, *t*, *r*) with confidence intervals, effect sizes, degrees of freedom and *P* value noted<br>*Give P values as exact values whenever suitable.*
☒	☐	For Bayesian analysis, information on the choice of priors and Markov chain Monte Carlo settings
☐	☒	For hierarchical and complex designs, identification of the appropriate level for tests and full reporting of outcomes
☒	☐	Estimates of effect sizes (e.g. Cohen's *d*, Pearson's *r*), indicating how they were calculated

Our web collection on statistics for biologists contains articles on many of the points above.

Software and code

Policy information about availability of computer code

Data collection	Device control: LabVIEW (Version 11.0, National Instrument); Image acquisition: NIS-Element (Ver. 4.0); LabSolutions software (Shimadzu); Agilent 2100 Bioanalyser Expert Software (Agilent Technologies, Inc., USA); NovaSeq Control Software v1.6.0; Real Time Analysis (RTA) v3.4.4; bcl2fastq2 v2.20;
Data analysis	BowTie (v.2.1.0); STAR Aligner (2.7.1a); Ensembl ver.98; RSEM (ver. 1.3.0); DEseq2 (ver. 1.8.2); WEB-based Gene Set Analysis Toolkit (WebGestalt); Gene Set Enrichment Analysis (GSEA, ver.4.0.4); ggplot2 package; CellProfiler software (Broad Institute of Harvard and MIT, Version 3.1.9); Orange 3 software (Version 3.23.1; Bioinformatics Laboratory); R software (ver. 3.5.2; https://www.r-project.org/);

For manuscripts utilizing custom algorithms or software that are central to the research but not yet described in published literature, software must be made available to editors and reviewers. We strongly encourage code deposition in a community repository (e.g. GitHub). See the Nature Research guidelines for submitting code & software for further information.

Data

Policy information about availability of data

All manuscripts must include a data availability statement. This statement should provide the following information, where applicable:
- Accession codes, unique identifiers, or web links for publicly available datasets
- A list of figures that have associated raw data
- A description of any restrictions on data availability

The mRNA-seq data have been deposited in the NCBI Gene Expression Omnibus (GEO) under accession number GSE152091.

Field-specific reporting

Please select the one below that is the best fit for your research. If you are not sure, read the appropriate sections before making your selection.

☒ Life sciences ☐ Behavioural & social sciences ☐ Ecological, evolutionary & environmental sciences

For a reference copy of the document with all sections, see nature.com/documents/nr-reporting-summary-flat.pdf

Life sciences study design

All studies must disclose on these points even when the disclosure is negative.

Sample size	Data were analyzed by using 3 groups of biological replicates.
Data exclusions	No data were excluded.
Replication	Cell culture experiments were repeated on 3 groups.
Randomization	The samples were not randamized.
Blinding	Investigators were not blinded to group allocation.

Reporting for specific materials, systems and methods

We require information from authors about some types of materials, experimental systems and methods used in many studies. Here, indicate whether each material, system or method listed is relevant to your study. If you are not sure if a list item applies to your research, read the appropriate section before selecting a response.

Materials & experimental systems

n/a	Involved in the study	
☒	☐	Antibodies
☐	☒	Eukaryotic cell lines
☒	☐	Palaeontology and archaeology
☒	☐	Animals and other organisms
☒	☐	Human research participants
☒	☐	Clinical data
☒	☐	Dual use research of concern

Methods

n/a	Involved in the study	
☒	☐	ChIP-seq
☒	☐	Flow cytometry
☒	☐	MRI-based neuroimaging

Eukaryotic cell lines

Policy information about cell lines

Cell line source(s)	HepG2 hepatocellular carcinoma and Caco-2 human colorectal adenocarcinoma cell lines were obtained from the American Type Culture Collection.
Authentication	All the cell lines were authenticated by the American Type Culture Collection before distribution to us.
Mycoplasma contamination	All the cell lines were tested for mycoplasma contamination at the American Type Culture Collection.
Commonly misidentified lines (See ICLAC register)	No commonly misidentified cell lines were used.
